# Deep evolutionary conservation of a sex-determining locus without sequence homology

**DOI:** 10.1073/pnas.2522417123

**Published:** 2026-01-05

**Authors:** Chuanxin Yu, Dean Hodapp, Safira Moog, Simon Dupont, Eric Darrouzet, Claudia Isabelle Keller Valsecchi, Thomas Joseph Colgan, Qiaowei Pan, Hugo Darras

**Affiliations:** ^a^Max Planck Institute for Biology, Tübingen 72076, Germany; ^b^Institute of Organismic and Molecular Evolution, Johannes Gutenberg University, Mainz 55128, Germany; ^c^Institut de Recherche sur la Biologie de l’Insecte (UMR 7261) CNRS, University of Tours, Tours 37200, France; ^d^Institute of Molecular Biology, Mainz 55128, Germany; ^e^Biozentrum, University of Basel, Basel 4056, Switzerland; ^f^Center for Evolutionary and Organismal Biology, School of Medicine, Zhejiang University, Hangzhou 310058, China

**Keywords:** evolution, hymenoptera, sex determination, comparative genomics

## Abstract

Sex determination is fundamental to eukaryotic life, yet its molecular basis varies widely across the tree of life. In most animal clades, sex-determining mechanisms are highly diverse and evolve rapidly. Here, we identify an exception in aculeate Hymenoptera, an ancient and diverse clade of haplodiploid insects that includes ants, bees, and stinging wasps. By integrating comparative genomics across Hymenoptera with genetic mapping in bumblebees and hornets, we reveal that the ANTSR locus, a multiallelic noncoding locus, has been maintained for over 150 My as the primary instructive signal for female development. This locus is located in a conserved synteny block that originated at the base of Aculeata and functions as a highly polymorphic, zygosity-based sex determiner, with only heterozygous individuals developing as females across lineages. Despite its deep evolutionary conservation, this sex locus shows no detectable sequence homology among lineages. These findings demonstrate that an essential noncoding locus can retain its function over deep evolutionary time without sequence conservation. More broadly, our results highlight haplodiploid insects as a powerful model for studying the evolution of sex determination mechanisms beyond those linked to sex chromosomes.

## Significance

Sex determination is fundamental to eukaryotic development, yet its molecular mechanisms are remarkably labile, especially in insects. Studying Hymenoptera, the order that includes ants, bees, and wasps, we identified an exception: a primary sex-determining locus conserved for over 150 My. This multiallelic noncoding locus consistently determines female development when heterozygous. Despite deep functional conservation, this locus shows no detectable sequence similarity across species. These findings challenge the prevailing view that insect sex determination evolves rapidly and provide a rare example of long-term functional conservation despite the absence of DNA sequence similarity.

The existence of two sexes is a deeply conserved feature of eukaryotic life; however, the mechanisms that specify sexual fate during embryonic development are highly diverse and evolve rapidly (1, 2). In animals, only two master sex determination genes, *Sry* in mammals and *Dmrt1* in birds, are known to have persisted over long evolutionary timescales (greater than 100 My) (3–5). In contrast, other lineages, particularly insects, show frequent turnover of the primary signals that initiate sex determination (6). Until recently, nine primary sex-determining genes have been identified in insects: *sxl*, *moy,* and *Mdmd* in flies (7–9), *nix* and *yob* in mosquitoes (10, 11), *fem* piRNA in the silkworm (12), *csd* in honeybees (13), *wom* in the wasp *Nasonia vitripennis* (14), and lncRNA *ANTSR* (pronounced “ant-ser”) in ants (15). All except *ANTSR,* whose evolutionary origin remained unclear, are restricted to recent lineages and converge on regulating the splicing and expression of the conserved effectors *doublesex* and, in most species, *transformer* (16, 17). This recurring pattern of evolutionary short-lived master regulators and conserved terminal effectors aligns with Wilkins’ original conceptual framework of sex determination evolution (18), which applies broadly across animals and posits that sex determination pathways evolve from the bottom up, through successive recruitment of new upstream regulators.

In Hymenoptera (ants, bees, wasps, and sawflies), females develop from fertilized, diploid eggs, while males arise from unfertilized, haploid eggs (19). Occasionally, fertilized eggs produce diploid males, but these individuals are usually sterile (20). Interestingly, the three hymenopteran species where sex determination has been studied to date each exhibit fundamentally different mechanisms initiating female development. In *N. vitripennis*, sex is determined by maternal imprinting of the *wom* gene; only fertilized eggs carrying an active paternal copy develop as females (14). In contrast, the honeybee relies on the *csd* gene, which harbors more than 100 haplotypes reported across the species. Fertilized eggs with two different haplotypes form heterodimeric proteins that trigger female development, whereas haploid eggs and fertilized eggs homozygous at *csd* develop as males (13, 21). Both *wom* and *csd* conform to the Wilkins model as recently evolved genes restricted to *Nasonia* wasps and honey bees (14, 22). In the Argentine ant, *Linepithema humile*, we recently identified a complementary sex determination system analogous to that of the honeybee, which operates through a distinct molecular mechanism. In this system, sex is determined by genotype at a 5 kb highly polymorphic noncoding region downstream of the long noncoding RNA *ANTSR,* containing seven distinct alleles (15). Eggs that are heterozygous at this locus show strong *ANTSR* expression and develop as females, whereas hemizygous (haploid) or homozygous eggs show low expression and develop as males. As the mechanism by which genotype influences *ANTSR* expression remains unclear, we here provisionally use the term “ANTSR locus” (not italicized) to denote the genomic region encompassing the lncRNA *ANTSR* and the downstream polymorphic sex-determining region. Syntenic regions corresponding to the ANTSR locus also display sex-specific heterozygosity in *Ooceraea biroi* (23) and *Vollenhovia emeryi* (24), two other formicoid ants, suggesting that this locus represents the ancestral sex-determining region of the formicoid clade, which originated about 125 Mya (25). However, without broader comparative evidence, this pattern could also reflect the independent recruitment of the same genomic region in three separate lineages, as frequently observed for sex-determining genes (26).

Here, by integrating comparative genomic evidence across the Hymenoptera phylogeny, we found that the ANTSR locus has governed sex determination across Aculeata (ants, bees, and stinging wasps) for over 150 My, revealing a rare case of long-term evolutionary stability of a sex determination system within one of the most diverse animal lineages. This locus has maintained its sex determining role despite complete sequence divergence, with no detectable homology across distant lineages. In parallel to our work, two recent studies reported sex-specific heterozygosity near the ANTSR locus in mason bees (27) and bumblebees (28), adding independent support for a conserved role of this region in Aculeata sex determination. Together, these findings demonstrate that noncoding elements controlling essential developmental processes can remain functionally conserved even after all sequence similarity has been lost.

## Results

**Fig. 1.**
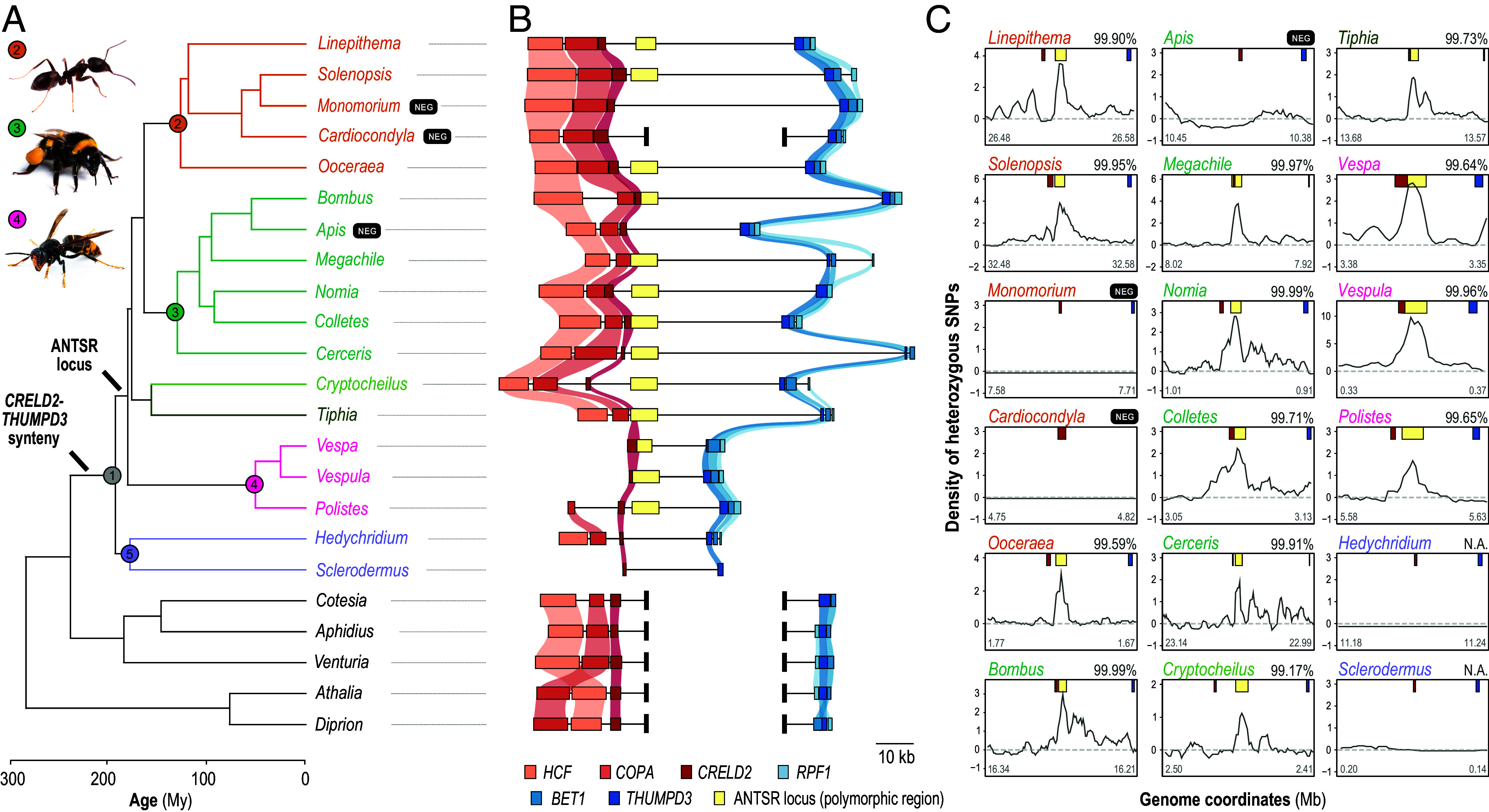
Genomic signatures reflecting long-term functional conservation of the ANTSR locus across 150 My of divergence. (*A*) Phylogeny constructed by reconciling subtrees from Peters et al. (31) and Romiguier et al. (25), with divergence times taken from Peters et al. Representative species used for each genus are, in order of appearance in the tree: *L. humile, S. invicta, M. pharaonis, C. obscurior, O. biroi, B. terrestris, A. mellifera, M. willughbiella, N. melanderi, C. gigas, C. rybyensis, C. praepositus, T. femorata, V. velutina nigrithorax, V. vulgaris, P. fuscatus, H. roseum, S.* “alternatusi,” *C. glomerata, A. gifuensis, V. canescens, A. rosae*, and *D. similis* (*SI Appendix*, Table S1). Colored disks denote: 1, Aculeata; 2, ants; 3, bees; 4, vespids; 5, Chrysidoidea. (images: Eric Isselée and Brais Seara, Adobe Stock). (*B*) Synteny analysis reveals that the 17 to 73 kb *CRELD2*–*THUMPD3* block containing the ANTSR locus originated approximately 160 to 200 Mya. Colored boxes represent annotated protein-coding genes and the candidate polymorphic sex-determining region of the ANTSR locus (see legend of panel *C*). Thick vertical bars indicate points of synteny disruption. (*C*) In most nonchrysidoid *Aculeata* species, the density of heterozygous SNPs in single-female genomes consistently peaks between the flanking genes *CRELD2* (red boxes) and *THUMPD3* (blue boxes), suggesting that this region emerged as a complementary sex-determining locus more than 150 Mya. Yellow boxes mark the boundaries of the three most heterozygous 5 kb windows within these peaks, delineating the location of the candidate multiallelic sex-determining region of the ANTSR locus. For each species, we report the percentile rank of the most heterozygous of these windows, calculated from the genome-wide distribution of heterozygosity across all 5 kb windows, and shown as a percentage (*SI Appendix*, Fig. S1). Heterozygosity values are scaled to each genome’s mean, depicted as a horizontal dashed line (see Methods). Three negative-control (marked by black “NEG” boxes) species that do not use the ANTSR locus for sex determination are included for comparison (*SI Appendix*, Text S1).

We previously identified the sex-determining ANTSR locus in the ant *L. humile* and investigated its evolutionary origin by performing BLAST searches. These analyses revealed no hits beyond closely related species (15). However, this pattern does not preclude conservation of this sex locus through genomic position rather than sequence identity (29, 30). To explore this possibility, we compared synteny flanking the ANTSR locus across 41 species spanning the order Hymenoptera (*SI Appendix*, Table S1). In *L. humile*, this locus is located between the protein-coding genes *CRELD2* and *THUMPD3*. Our results show that this gene block originated approximately 160 to 200 Mya in the last common ancestor of ants, bees, and stinging wasps (Aculeata), suggesting long-term conservation of this sex locus’s genomic context (Fig. 1 *A* and *B*). Between the flanking *CRELD2* and *THUMPD3* genes, no protein-coding genes are annotated in any aculeate genome. However, in 13 aculeate species other than *L. humile*, we identified either NCBI RefSeq annotations or spliced read evidence suggesting the presence of a candidate *ANTSR* lncRNA ortholog based on synteny (*SI Appendix*, Table S1).

We then investigated whether the syntenic region of the *Linepithema* ANTSR locus is associated with sex determination across Aculeata. In contrast to sex-chromosome systems, complementary sex determination systems, such as those in *L. humile* and the honeybee, maintain multiple haplotypes through negative frequency-dependent selection, as homozygosity at the sex locus results in the production of diploid males with reduced fitness (15, 32). In *L. humile*, the 5 kb sex-determining region downstream of lncRNA *ANTSR* is the most polymorphic in the genome, with heterozygous SNP density in individual females that can reach the top 0.1% genome-wide (Fig. 1*C*). We hypothesized that if the same locus mediates complementary sex determination in other species, females should also carry two divergent haplotypes, creating a localized peak in heterozygosity. Whole-genome resequencing data from females of 17 aculeate species (*SI Appendix*, Table S2) displayed this exact pattern. All species, except representatives of the Chrysidoidea superfamily (*Hedychridium roseum* and *Sclerodermus alternatusi*), showed a sharp peak of heterozygosity downstream of *CRELD2* (Fig. 1*C* and *SI Appendix*, Fig. S1), consistently ranking among the top 0.9% most polymorphic genomic segments. The specificity of this signal was further highlighted by the absence of elevated heterozygosity in species that either do not rely on a complementary sex determination system, such as the inbreeding ants *Monomorium pharaonis* and *Cardiocondyla obscurior* (33), or rely on alternative molecular mechanisms, such as honeybees, which depend on the lineage-specific *csd* gene (13, 34) (*SI Appendix*, Text S1). Taken together, these observations suggest that *L. humile*’s ANTSR locus, located between *CRELD2* and *THUMPD3*, gained a sex-determining role early in aculeate evolution, probably soon after Chrysidoidea diverged from the remaining aculeates.

To test our prediction, we generated inbred crosses and population genomic data from bumblebees and hornets, two aculeate lineages that, together with ants, span more than 150 My of evolution.

**Fig. 2.**
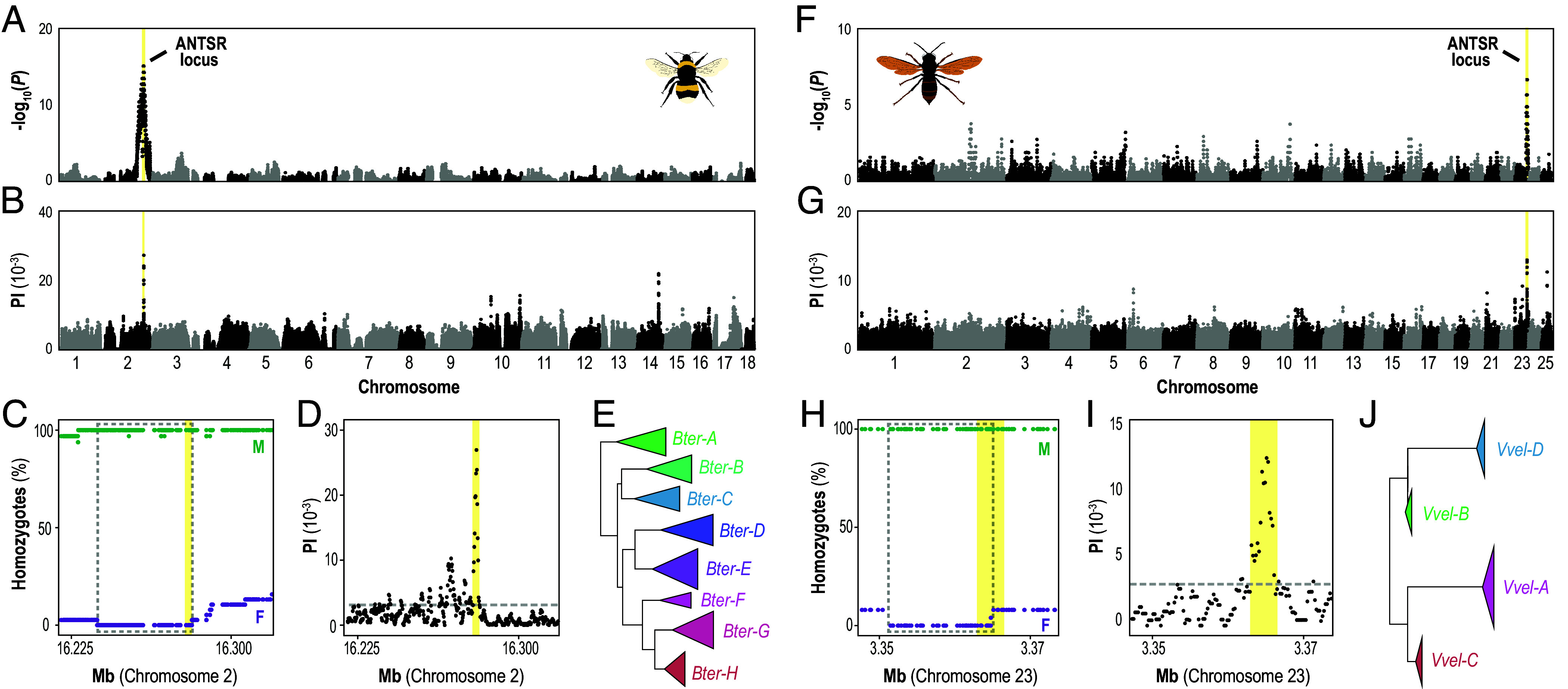
Genetic mapping in bumblebees and hornets identifies the ANTSR locus as the ancestral complementary sex determination locus of Aculeata. (*A*) Comparison of SNP heterozygosity between *Bombus terrestris* females (N = 34) and diploid males (N = 29) from nine inbred crosses (*SI Appendix*, Tables S3 and S4). Negative log *P*-values from two-proportion Z-tests are plotted against genomic coordinates; the position of the ANTSR locus is highlighted in yellow (*SI Appendix*, Fig. S4). (*B*) Nucleotide diversity (PI) in 1-kb windows across the *B. terrestris* genome based on 84 field-collected haploid males (*SI Appendix*, Table S5). (*C*) Percentage of *B. terrestris* females (F, purple) and diploid males (M, green) that are homozygous at the candidate sex locus. The dashed box marks the genomic interval consistently heterozygous in females and homozygous in diploid males (Chromosome 2: 16,237,070 to 16,281,509). (*D*) Zoomed view of panel *B*. The yellow shaded area highlights the candidate multiallelic sex-determining region (16,278,001 to 16,282,201 bp), defined as the region where all windows rank within the top 1% most diverse in the genome (gray dashed line). (*E*) Phylogenetic tree of haplotypes from the multiallelic sex-determining region of the ANTSR locus in *B. terrestris* based on 84 field-collected haploid individuals, revealing eight haplogroups (*SI Appendix*, Fig. S4; region highlighted in *D*). (*F*) Comparison of SNP heterozygosity between wild *Vespa velutina nigrithorax* females (N = 25) and diploid males (N = 7) from 30 colonies (*SI Appendix*, Fig. S6). (*G*) Nucleotide diversity (PI) in 1-kb windows across the *V. velutina nigrithorax* genome (N = 25 females; *SI Appendix*, Table S6). (*H* and *I*) Zoomed view of the sex locus in *V. velutina nigrithorax*, showing genomic intervals with sex-specific differences in heterozygosity (*H*; Chromosome 23: 3,351,490 to 3,364,744) and high polymorphism (*I*; 3,362,601 to 3,366,601). See also panels *C* and *D* legends. (*J*) Phylogenetic reconstruction of allele from the sex-determining region in *V. velutina nigrithorax*, based on seven diploid males and inferred phased haplotypes from 25 females, revealed four distinct haplogroups within the invasive French population (*SI Appendix*, Fig. S6). Because intrahaplogroup diversity could not be fully resolved from the inferred haplotypes, branch depths within collapsed clades are shown for visualization purposes only.

For the buff-tailed (large earth) bumblebee, *Bombus terrestris*, we performed brother-sister crosses to obtain diploid males. We expected that half of the crosses, in which queens shared the same maternal allele at the sex locus as their brother, would produce diploid males during the early colony phase, when usually only workers are produced (35, 36). Eighteen of the 30 sib-mated queens produced early males (two-tailed binomial test against a 50% expectation, *P* = 0.36). Genome sequencing of males from nine of these queens confirmed that most were diploid: eight queens produced only diploid males (N = 26), while one queen had both diploid and haploid males (N = 2 each; *SI Appendix*, Figs. S2 and S3 and Tables S3 and S4). Comparing heterozygosity between these diploid males and their sisters identified a single 44.4 kb genomic window on chromosome 2 (NC_063270.1), encompassing the candidate location of the ANTSR locus, that is consistently heterozygous in females but homozygous in diploid males (two-proportion Z tests, all SNPs *P* < 2.2 × 10^−16^; Fig. 2 *A* and *C* and *SI Appendix*, Fig. S4 and Table S4), confirming that this region determines female sex through heterozygosity. To further refine the boundaries of this multiallelic sex determining region, we analyzed whole-genome sequencing data from bees collected in Great Britain and Ireland (37, 38). This analysis identified a 4.2 kb window between *CRELD2* and a provisionally annotated gene, which we inferred to be the lncRNA *ANTSR*, as the most polymorphic region across the entire genome (Fig. 2 *B* and *D*). Eight distinct haplotypes of this region coexist within and across populations (Fig. 2*E* and *SI Appendix*, Figs. S4 and S5 and Table S5), closely resembling the polymorphism observed at the sex locus of *L. humile* (15). These findings demonstrate that the region neighboring the lncRNA *ANTSR* functions as a multiallelic sex-determining locus in both ants and bumblebees, confirming our predictions.

We further extended our analyses to the Asian hornet, *Vespa velutina nigrithorax*, where diploid male production is frequent in the invasive French population (39). Analysis of field-collected diploid males and females revealed a single 13.3 kb interval on chromosome 23 (NC_062210.1), encompassing the candidate location of the ANTSR locus, that was consistently heterozygous in females but homozygous in diploid males (two-proportion Z test, all SNPs *P* < 2.2 × 10^−6^; Fig. 2 *F* and *H* and *SI Appendix*, Fig. S6 and Table S6). Nucleotide diversity within this interval reached its genome-wide maximum in a 4 kb window, whose boundaries overlap with *CRELD2* and the putative location of the *ANTSR* lncRNA transcript, supported by the presence of mapped spliced RNA reads (Fig. 2 *G* and *I* and *SI Appendix*, Table S1). We identified only four distinct haplotypes in France, a restricted number that likely contributes to the high incidence of diploid males in this invasive population (Fig. 2*J* and *SI Appendix*, Figs. S5 and S6). Analyzing the population genomic data from an additional *Vespa* species provided further validation of the conservation of this locus across hornets. Among more than 100 *Vespa mandarinia* individuals sequenced by Taylor et al. (40), we identified two males whose genome-wide heterozygosity patterns indicate diploidy (*SI Appendix*, Fig. S7). In this species, the candidate location of the multiallelic sex-determining region, situated between *CRELD2* and *THUMPD3,* was once again the genomic region exhibiting the most significant difference in heterozygosity between females and diploid males, and it contained the highest nucleotide diversity in the genome (*SI Appendix*, Figs. S8 and S9). These convergent patterns across species further support the ANTSR locus as the ancestral sex-determining locus in Aculeata.

**Fig. 3.**
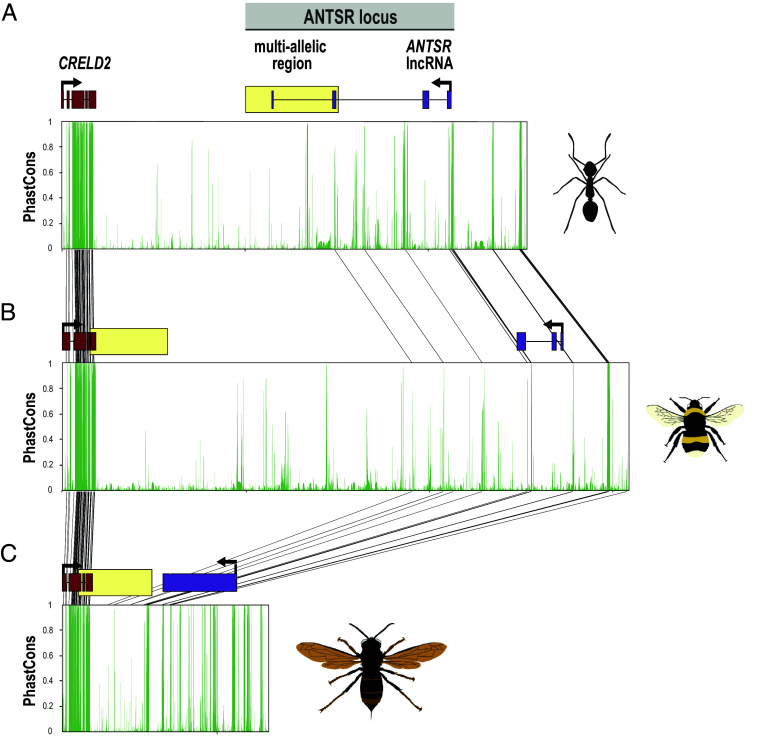
Absence of sequence homology for lncRNA *ANTSR* and the downstream multiallelic sex-determining region across Aculeata. PhastCons conservation scores, based on a multispecies alignment of 13 aculeate species with the ANTSR locus as the candidate sex-determining locus (Fig. 1), are shown for the three species where this sex locus has been confirmed through genetic mapping: (*A*) *Linepithema humile* (15), (*B*) *Bombus terrestris*, and (*C*) *Vespa velutina nigrithorax* (this study). No sequence conservation signal is observed across these species. Dark gray lines connecting the three panels indicate aligned positions with PhastCons scores greater than 0.5, which we considered “conserved.” These sites are primarily restricted to the protein-coding exons of *CRELD2*. Boxes indicate the positions of *CRELD2* exons (red), lncRNA *ANTSR* exons (purple; “unannot.” = gene model not resolved in *V. velutina nigrithorax*, see *SI Appendix*, Table S1), and the candidate downstream multiallelic sex-determining region (yellow box defined in *L. humile* from Pan et al. (15); in *B. terrestris* and *V. velutina nigrithorax* based on intervals where all windows fall within the top 1% of genome-wide polymorphism, see Fig. 2).

Given that the ANTSR locus is conserved in sex-determining function across species, we next asked whether any sequence-level homology was retained that could be missed when searching with BLAST. To improve sensitivity, orthologous regions from 13 aculeate species were aligned using a phylogeny-aware method (41), and phastCons scores, which measure evolutionary conservation across species by accounting for their phylogenetic relationships, were computed. Neither lncRNA *ANTSR* nor its downstream multiallelic region showed conservation across Aculeata (Fig. 3). To account for the possibility of missing homologous sequences when analyzing a single haplotype of the multiallelic sex-determining region per species, we also compared each of the seven well-characterized *L. humile* haplotypes with the reference genomes of *B. terrestris* and *V. velutina nigrithorax*. No additional similarity was detected (*SI Appendix*, Fig. S10). Altogether, these results indicate that this ancient sex locus lacks detectable sequence similarity across Aculeata, despite its conservation in genomic position and function.

## Discussion

Our genetic mapping demonstrates that the ANTSR locus, first described in the Argentine ant (15), later mapped in the ant *O. biroi* (23), and also linked to sex-specific heterozygosity in the ant *V. emeryi* (24), governs sexual development in bumblebees and hornets. Analysis of female heterozygosity patterns suggests that this locus has retained a sex-determining function across major lineages of the Aculeata but is absent from the basal Chrysidoidea lineage. These findings support a single origin 150 to 170 Mya (31) and persistence across one of the most extensive radiations in the animal kingdom. While this manuscript was in review, two independent studies by Rönneburg et al. (27) and Leung et al. (28) reported sex-specific heterozygosity in the region spanning the ANTSR locus in bumblebees and mason bees, further supporting its widespread role across Aculeata. The ANTSR locus represents an exceptional case of long-term stability among animal sex-determining systems. Until now, only two such systems were known to have persisted over 100 My: *Sry* in therian mammals (~160 My) and *Dmrt1* in birds (~140 My) (3–5). In other taxa with differentiated sexes, sex-determining loci have been shown to evolve rapidly under the influence of newly arising sexually antagonistic alleles and the accumulation of deleterious mutations on nonrecombining sex chromosomes (2, 42). By contrast, complementary sex determination systems in haplodiploid organisms rely on alleles shared by both sexes and show only localized recombination suppression around the sex locus (43). These characteristics likely explain the long-term stability of the ANTSR locus compared with sex loci linked to entire chromosomes and restricted to one sex.

Despite the stability of the ANTSR locus, a few losses have occurred in the Aculeata. In honey bees, this ancestral sex locus was replaced by the *csd* gene, which also acts through allelic complementarity but via a distinct mechanism (13, 21). This turnover may reflect degeneration of the ancestral locus caused by the accumulation of deleterious mutations within its nonrecombining alleles (44). In the inbred ants *C. obscurior* and *M. pharaonis*, sex appears to rely on mechanisms independent of allelic diversity, possibly analogous to the imprinting-based system described outside of the Aculeata in the wasp *Nasonia* (14, 33) (*SI Appendix*, Text S1). These independent transitions may reflect selection for sex determination systems that remain functional under conditions of low genetic diversity (45).

In many biological systems, gene function depends on interactions between distinct alleles, such that only heterozygotes produce viable outcomes. This selective pressure promotes the maintenance of high allelic diversity. Classic examples of such systems include the self-incompatibility S-locus in flowering plants and the *csd* gene in honeybees, which harbor dozens to hundreds of haplotypes (44, 46, 47). Strikingly, the multiallelic region of the ANTSR locus exhibits comparatively low allelic diversity in all three examined aculeate species. We previously documented seven haplotypes in French populations of the invasive ant *L. humile* (15), whereas the Asian hornet populations examined here contained only four haplotypes. Although these values could reflect diversity losses following colonization bottlenecks, native bumblebee populations were similarly impoverished, with only eight haplotypes across populations. Greater allelic diversity was expected, as populations with fewer than ten haplotypes are predicted to produce over 10% diploid males from fertilized eggs, which have low fitness and divert resources from female production (32). This suggests that the mechanism of this multiallelic region, unlike that of other multiallelic systems, imposes intrinsic constraints on the number of functionally distinct haplotypes it can sustain. Across divergent aculeate lineages, the *CRELD2–THUMPD3* interval harboring this region lacks protein-coding genes, yet RNA-seq data reveal lncRNAs likely orthologous to *L. humile*’s *ANTSR.* Future work should clarify how heterozygosity at this locus directs female development and inspect whether this signal is mediated by the nearby lncRNA through its expression level, as observed in *L. humile*.

Overall, our findings provide a clear example of a noncoding element with known origin and role. Together with independent studies published concurrently in bees (27, 28), our study reveals the long-term conservation of this unusual sex-determining locus over more than 150 My and reinforces the growing recognition that functional noncoding elements, such as regulatory regions, can persist over long evolutionary timescales without detectable sequence homology, and thus escape detection by sequence-based methods (29, 48). Moreover, the identification of this broadly conserved sex-determining region provides an immediately deployable molecular sex marker for breeding and conservation initiatives to monitor sex-locus diversity within the Aculeata, a clade that encompasses essential pollinators, biological control agents, and invasive pests.

## Methods

### Reconstruction of the Evolutionary History of the ANTSR Locus.

We analyzed genome annotations from 41 hymenopteran species with well-assembled genomes on NCBI, showing no breaks near *CRELD2* and *THUMPD3*. For genera represented by multiple species, we selected one species (*SI Appendix*, Table S1). When RefSeq annotations were unavailable, gene positions were inferred using BLAST with *L. humile* transcripts as queries. Variant calling for 22 species using individual female resequencing data (*SI Appendix*, Table S2) was carried out by aligning reads with BWA v0.7.18 (49), removing PCR duplicates with sambamba markdup v1.2.1 (50), and calling variants with FreeBayes v1.0.2 (51) with clustering disabled. Low-quality variants were filtered using VCFtools v.0.1.16 (“*--minQ 20*”) (52). Indels were decomposed using vt’s command “*decompose_blocksub*” (53). Lowercase-annotated repetitive regions were hard-masked. Heterozygous sites were counted in 5-kb sliding windows with 1-kb steps across the genome, and the genomic window with the highest heterozygosity downstream of *CRELD2* was ranked for each species. To enable cross-species comparisons despite differences in baseline genetic diversity, the numbers of heterozygosity sites were normalized for each species using the transformation (x − μ) / (x_99_ − μ), where *x* is the number of heterozygous sites per window, *μ* is the genome-wide mean, and *x_99_* denotes the 99th percentile of the distribution.

### Sampling of Diploid Males and Females.

For the purposes of obtaining bumblebee queens, two *B. terrestris* colonies were obtained from the commercial supplier Koppert in the Netherlands. Colonies were fed ad libitum and maintained in an environmentally controlled room under red light illumination. Sexual production was monitored, and sib-matings were conducted using related virgin queens and males, with visual confirmation of mating (54). A total of 30 sib-mated queens were obtained and monitored for colony initiation and offspring emergence. Males were distinguished from female offspring based on anatomical features, including lack of aculeus, curved last abdominal tergite, and longer antennae. We selected one to four individuals of each sex for each of nine diploid-male-producing colonies (*SI Appendix*, Table S3). For *V. velutina nigrithorax*, females and diploid males were sourced from 30 colonies in France (*SI Appendix*, Table S6), with diploid male status confirmed by microsatellite genotyping (39, 55). DNA was extracted using either BioSprint DNA Animal or MagAttract DNA Kit (Qiagen), and two ng of DNA were used to prepare custom libraries with Tn5 transposase, 13 PCR cycles, and AMPure XP (Beckman) purification. The libraries were sequenced on a NovaSeq X Plus (PE 150 bp; *SI Appendix*, Table S4).

### Identification of Sex-Determining Locus.

To map the sex locus in *Bombus* and *Vespa*, reads were aligned to the latest RefSeq reference genome of each species (see *SI Appendix*, Table S7 for parameter details). Briefly, variants were called as described above, and high-quality biallelic SNPs were filtered with VCFtools v0.1.16 and bcftools v1.21 (56). We confirmed male diploidy by comparing genome-wide heterozygosity levels with those of diploid females, and we inferred runs of homozygosity in diploid individuals using PLINK v1.9. For each SNP, we calculated the proportion of homozygous individuals by sex and tested for differences between females and diploid males using a two-proportion Z-test in R v4.4.1. Nucleotide diversity was estimated from haploid male resequencing data in *Bombus* and diploid female data in *Vespa* using PopGenome v2.7.5 (57) with 1-kb sliding windows and 200-bp overlap. To assess haplotype diversity at the multiallelic sex-determining region of the ANTSR locus in *B. terrestris* and *V. velutina nigrithorax*, VCFs were converted to FASTA with vcf-kit v0.2.6 (58), and maximum-likelihood phylogenies were reconstructed in IQ-TREE v2.3.6 with ascertainment bias correction and SH-aLRT support (“*–st DNA –m GTR+ASC –bb 1000 –alrt 1000*”) (59). In *Vespa*, where only a few haploid males were available, diploid female genotypes were manually phased based on male allele combinations, with ambiguous inferences scored as missing (*SI Appendix*, Text S2).

### Sequence Conservation.

We aligned the *CRELD2-THUMPD3* region from 13 aculeate species using two iterations of PRANK v.170427 (“*-F -DNA -nomafft*”) (41), incorporating phylogenetic topologies from previous works (25, 31). A neutral substitution model was then fitted using phyloFit (PHAST v1.6.9) (60) under the SSREV model with symmetrical base frequencies, based on the chosen topology. Model parameters were initialized at random and then reoptimized in a second run, using the initial estimates as starting values. We calculated base-wise conservation scores using phastCons (61). To control for haplotype bias, we assembled the seven *L. humile* sex haplotypes de novo from published male Illumina reads (15) using SPAdes v3.15.2 (“*--isolate -k 21,33,55,77,99*”) (62). Each haplotype was then aligned to the *B. terrestris* and *V. velutina nigrithorax* reference genomes using LAGAN (63).

## Supplementary Material

Appendix 01 (PDF)

## Data Availability

All sequencing reads generated for this study are deposited in NCBI (BioProject: PRJNA1295409) (64) and are publicly available. Study data are included in the article and/or *SI Appendix*.
