# Supplementary material for: Deep evolutionary conservation of a sex-determining locus without sequence homology
Source: Proc Natl Acad Sci U S A. 2026 Jan 5;123(2):e2522417123. doi: 10.1073/pnas.2522417123 (PMC12799146; doi:10.1073/pnas.2522417123)
Supplement: Supplementary file 1 — Appendix 01 (PDF) [file pnas.2522417123.sapp.pdf]

PNAS

Supporting Information for

Deep evolutionary conservation of a sex-determining locus without sequence homology

Authors: Chuanxin Yu, Dean Hodapp, Safira Moog, Simon Dupont, Eric Darrouzet, Claudia Isabelle Keller Valsecchi, Thomas Joseph Colgan, Qiaowei Pan, Hugo Darras

Email: qiaowei.pan@tuebingen.mpg.de, hdarras@zju.edu.cn (co-corresponding authors)

This PDF file includes:

Supporting text
Figures S1 to S10
Tables S1 to S7
SI References

Supporting text

Text S1. Negative controls

We included four species known to either not rely on complementary sex determination or not use the ANTSR locus as the primary sex-determining signal as negative controls in our genomic analyses: *Apis mellifera, Apis cerana, Cardiocondyla obscurior,* and *Monomorium pharaonis*. The honeybees *Apis mellifera* and *Apis cerana* rely on the *csd* gene for complementary sex determination (1, 2). The gene *csd* is a multi-allelic, protein-coding gene that evolved recently within the honeybee lineage (3). It is unrelated to the ANTSR locus, which is located in a different genomic region and operates through a distinct molecular mechanism. In females, amino acid differences between *csd* variants in heterozygous individuals enable the formation of functional protein heterodimers, which allow access to the functional domain and thereby activate the female developmental pathway (4). The homodimer of the CSD protein in hemizygous or homozygous individuals is not functionally capable of initiating female development. Because the ANTSR locus has been replaced by *csd* in this lineage, we did not expect high heterozygosity at its expected position to be maintained in these two species of honeybees. On the other hand, the ants *C. obscurior* and *M. pharaonis* are widely used as laboratory models for social insect research due to their ability to breed in captivity under laboratory conditions. In *C. obscurior,* (5) reported the near-complete absence of diploid males after ten generations of brother–sister mating, suggesting that this species does not rely on complementary sex determination. Similarly, no reports of diploid males exist in *M. pharaonis*, despite this species having been subjected to sibling-mating inbreeding in multiple laboratories for decades (6). We therefore consider it reasonable to assume that *M. pharaonis* does not rely on complementary sex determination, as is the case with *C. obscurior*. Complementary sex determination is considered ancestral and widespread in Hymenoptera (7), and our analyses suggest that the non-complementary systems of these two ant species must have evolved recently from an ancestor that relied on the ANTSR locus. To date, the only well-characterized hymenopteran species without complementary sex determination is the non-aculeate wasp *Nasonia vitripennis*, where maternal silencing of the *wom* gene suppresses female development in haploid embryos (8). We did not include it as a negative control because it belongs to the Chalcidoidea superfamily, which diverged from Aculeata over 220 million years ago and, thus, lacks the *CRELD2–THUMPD3* synteny block that gave rise to the ANTSR locus (see Main Figure 1).

Text S2. Parental haplotype inferences in *Vespa velutina nigrithorax*

In the 25 females of *V. velutina nigrithorax* sequenced, we inferred the presence of four haplotypes for the polymorphic sex-determining region of the ANTSR locus (A, B, C, and D), resulting in six possible heterozygous combinations (Figure S6). Sequencing of seven diploid males confirmed that all and only these four distinct haplotypes were present. Haplotypes A, B, and C were sequenced with high coverage, allowing us to assemble high-quality reference haplotypes. Haplotype D occurred in only one of seven males, which had lower coverage. To obtain the reference sequence of D, we complemented the male-derived data with inferences from female genotypes that carry the D haplotype. With reference sequences for the four haplotypes segregating in the invasive French population established, we manually phased the parental haplotypes of the diploid females. To do so, we applied the following rules for each SNP (reference genome alleles are noted R, alternative alleles A, and missing data N):

1. Homozygous female SNP genotype (A/A or R/R)
    1.1. If both parental candidates match the homozygous allele, assign that allele to both parents.
    1.2. If one candidate is missing (N), assign N to the corresponding parent.
    1.3. Otherwise, assign N to both parents.

2. Heterozygous female SNP genotype (A/R)
    2.1. If each allele matches one of the reference haplotypes, assign the matching allele to the corresponding parent and the alternative allele to the other parent.
    2.2. If assignments are ambiguous, encode both parental alleles as N.

3. Completely missing SNP genotype (N/N)
    3.1. Assign N to both maternal and paternal alleles.

For phylogenetic reconstruction, each homozygous male genotype was represented by two separate FASTA sequences, while the two phased haplotypes inferred from each female were used.

Figures

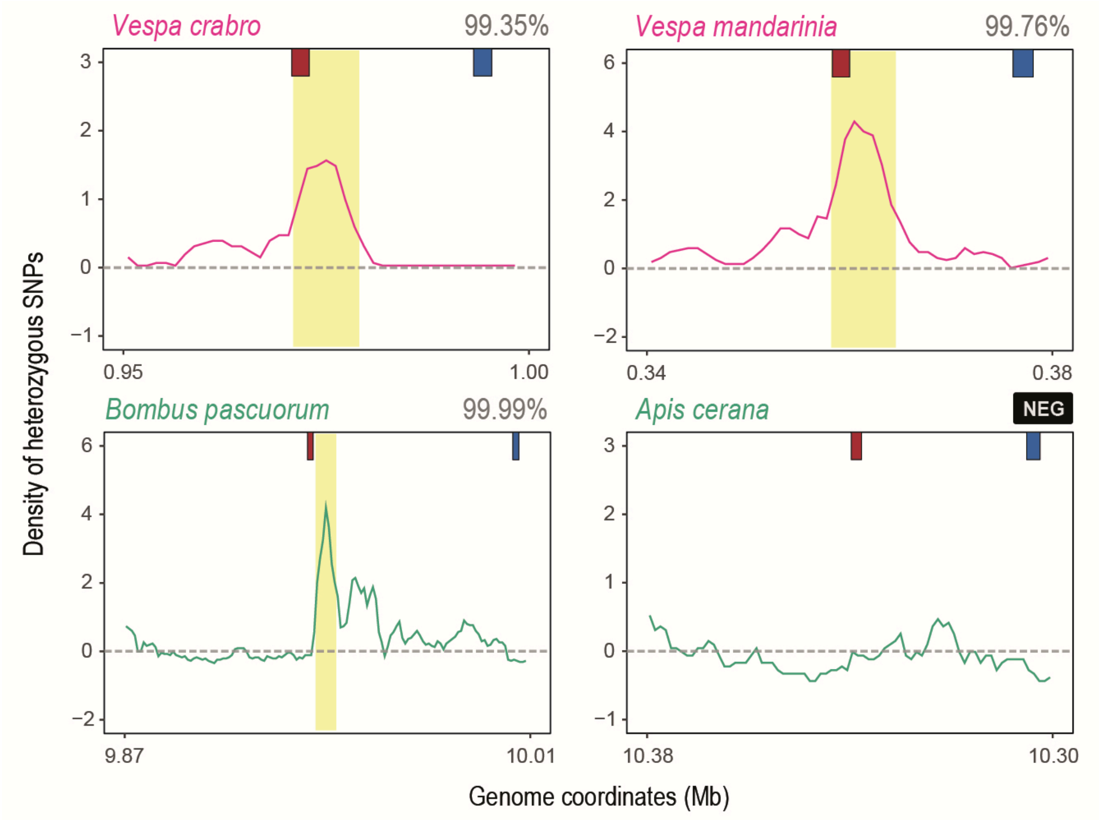

Figure S1. Heterozygous SNP density in single-female whole-genome sequences from four additional Hymenoptera species.

In *Bombus* and *Vespa* species, a localized peak of heterozygosity is consistently observed downstream of *CRELD2* (red boxes) and upstream of *THUMPD3* (blue boxes), marking the position of the multi-allelic sex-determining region of the ANTSR locus (yellow boxes, delineating the boundaries of the three most heterozygous 5 kb windows). The honeybee *Apis cerana*, known to use a different gene for sex determination, serves as a negative control (Text S1). Heterozygosity values were normalized within each species using the genome-wide mean (dashed line) and 99th percentile (see Methods). For each species, we report the percentile rank (expressed as a percentage) of the most heterozygous 5 kb window downstream of *CRELD2*, calculated from the genome-wide distribution of heterozygosity across all 5 kb windows.

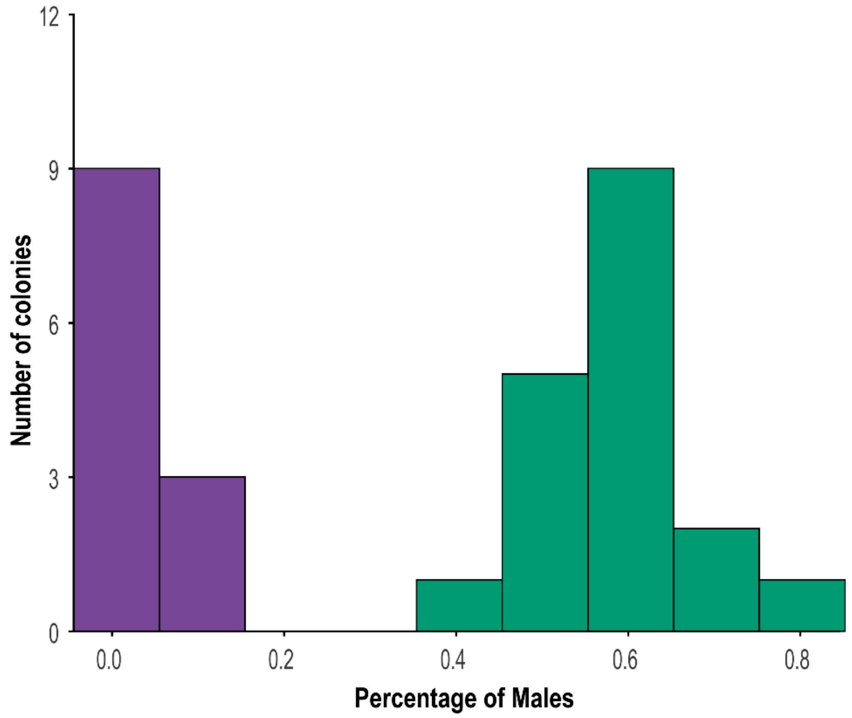

Figure S2. Early male production in *Bombus terrestris* colonies headed by sib-mated queens.

Histogram showing the number of colonies (y-axis) with varying proportions of males in the early brood, assessed three months after the onset of egg-laying—a period during which colonies typically produce only female workers. Data are from 30 colonies headed by sib-mated queens. Colonies classified as early-male-producing (producing >1 male) are shown in green; worker-producing colonies, which produced exclusively or nearly exclusively female workers (≤1 male), are shown in purple. See also Table S3.

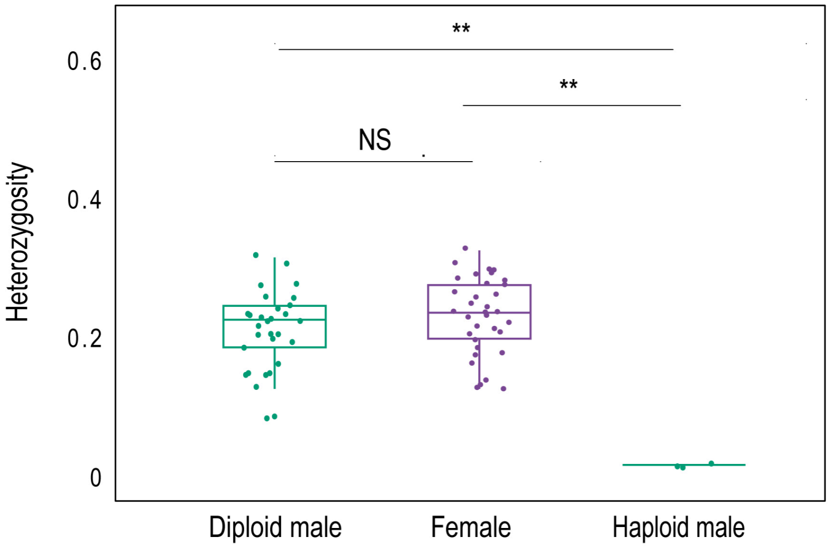

Figure S3. Genome-wide heterozygosity confirms the diploid status of males from early-male-producing *Bombus terrestris* colonies.

Comparison of genome-wide heterozygosity levels between diploid females (N = 34), diploid males (N = 29), and haploid males (N=3), all obtained from laboratory offspring of sib-mated queens (Table S3). Males from early-male-producing colonies exhibit heterozygosity levels comparable to females, confirming their diploid status and origin from fertilized eggs. No significant difference in heterozygosity was observed between diploid females and diploid males. In contrast, the heterozygosity level is significantly different between haploid males and diploids (Kruskal-Wallis test followed by pairwise Wilcoxon test with Bonferroni–Holm correction, ** = $P < 0.01$, NS = $p > 0.05$).

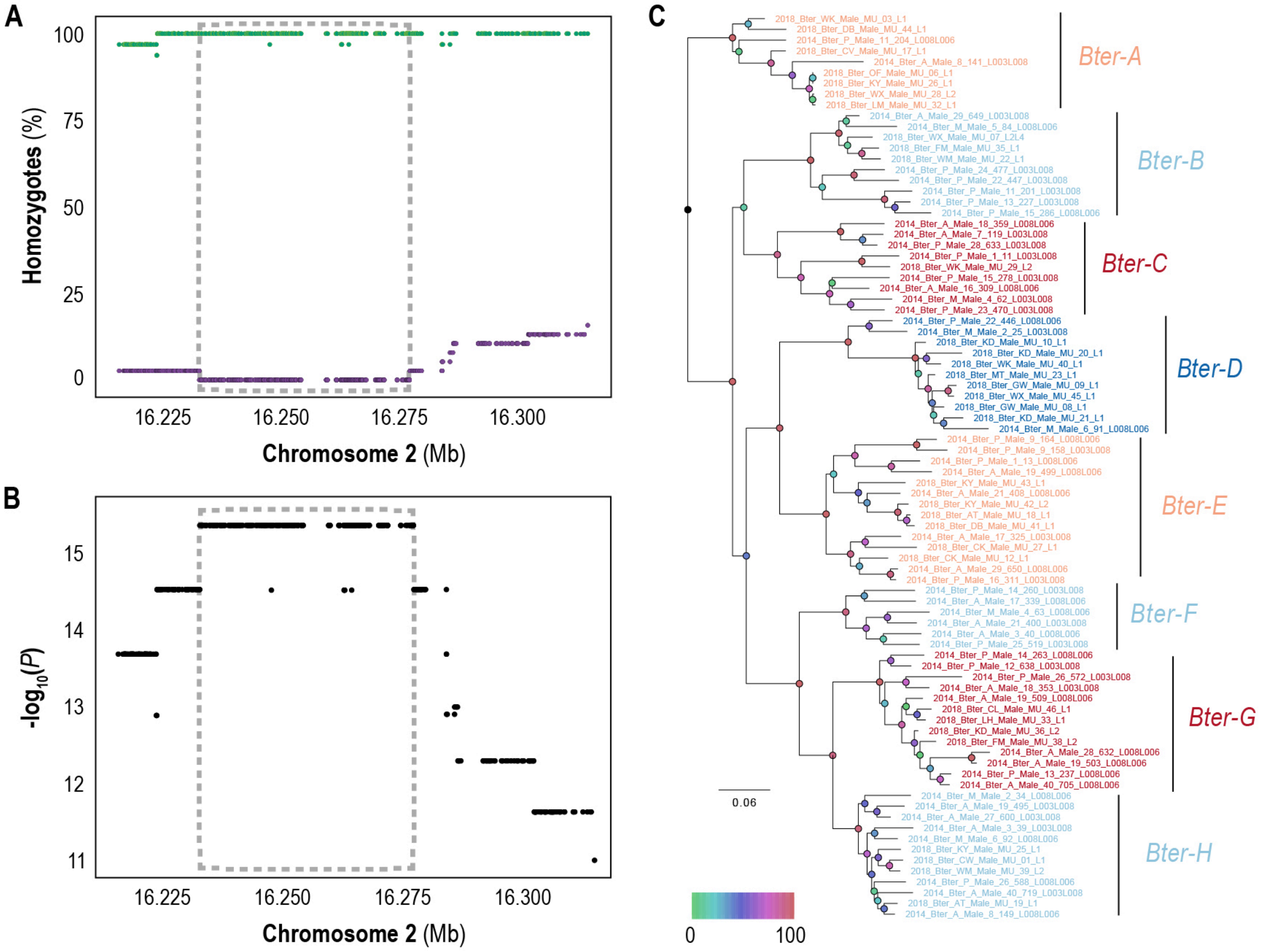

Figure S4. Genetic diversity around the ANTSR locus on chromosome 2 in *Bombus terrestris*.

A. Percentage of individuals with a run of homozygosity at each SNP position, shown separately for females (purple, N = 34) and diploid males (green, N = 29). The dashed box marks the genomic interval consistently heterozygous in females and homozygous in diploid males (Chromosome 2: 16,237,070–16,281,509). **B.** Negative log *P*-values from two-proportion *Z*-tests comparing homozygosity between females and diploid males (same dataset as in A). **C.** Maximum likelihood phylogenetic tree of haplotypes from the highly polymorphic region of the ANTSR locus using 950 single-nucleotide polymorphisms on chromosome 2 (16,278,001–16,282,201 bp). The dataset comprised 33 haploid males from Ireland and 51 from Great Britain (9, 10), revealing eight distinct haplogroups (*Bter-A* to *Bter-H*).

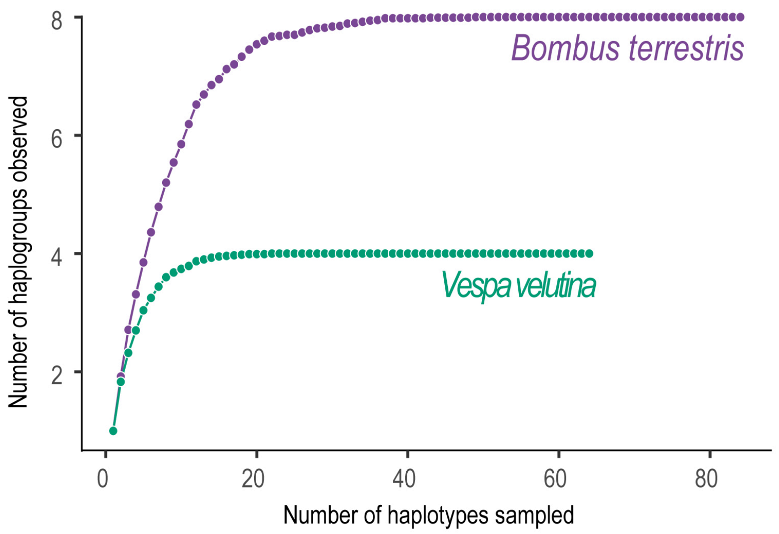

Figure S5. Rarefaction curves of haplotype discovery in field-collected haploid males of *Bombus terrestris* and field-collected *Vespa velutina nigrithorax* of the polymorphic region of the ANTSR locus. Cumulative number of unique haplotypes of the polymorphic region of the ANTSR locus detected as a function of individuals sampled, based on 33 *B. terrestris* haploid males from Ireland and 51 from Great Britain (9, 10), and 25 field-collected females and seven diploid males of *V. velutina nigrithorax*. Diploid genotypes of *V. velutina nigrithorax* were phased, resulting in 64 haploid sequences (Text S2). Rarefaction curves were generated using 100 random permutations, sampling haplogroups without replacement and recording the cumulative number of unique haplogroups at each step. Curves represent the average number of haplogroups observed across all permutations. Analyses were conducted separately for each species. The curves reach saturation, indicating that all haplotypes were recovered.

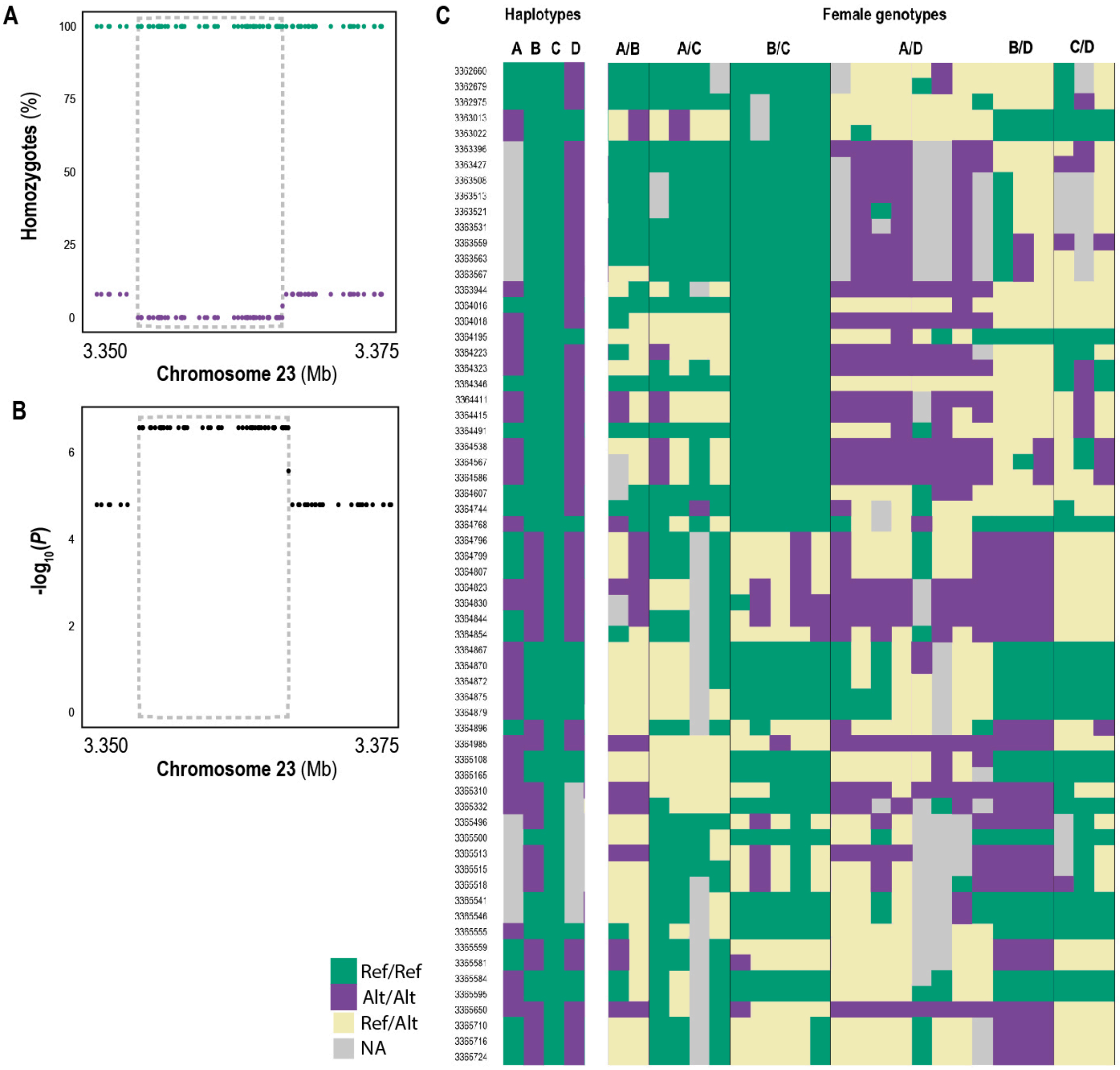

Figure S6. Genetic diversity around the ANTSR locus on chromosome NC_062210.1 in *Vespa velutina nigrithorax*

A. Percentage of individuals with a run of homozygosity at each SNP position, shown separately for females (purple, N = 25) and diploid males (green, N = 7). The dashed box marks the genomic interval consistently heterozygous in females and homozygous in diploid males (Chromosome 23: 3,351,490–3,364,744). **B.** Negative log *P*-values from two-proportion *Z*-tests comparing homozygosity between females and diploid males (same dataset as in A). **C.** Genotypes of the four haplotypes from the highly polymorphic region of the ANTSR locus (*Vvel-A* to *Vvel-D*), inferred from seven diploid male genotypes, and their six heterozygous combinations observed in 25 diploid females. Each column represents an individual, and each row corresponds to a single nucleotide polymorphism within the ANTSR locus on chromosome 23 (genomic position shown in the first column). Homozygous reference genotypes (Ref/Ref), corresponding to the genomic reference sequence, are shown in green; homozygous alternative genotypes (Alt/Alt; variants differing from the reference) are shown in purple; and heterozygous genotypes (Ref/Alt) are shown in yellow. Missing data, due to low coverage or large indels, are shown in grey.

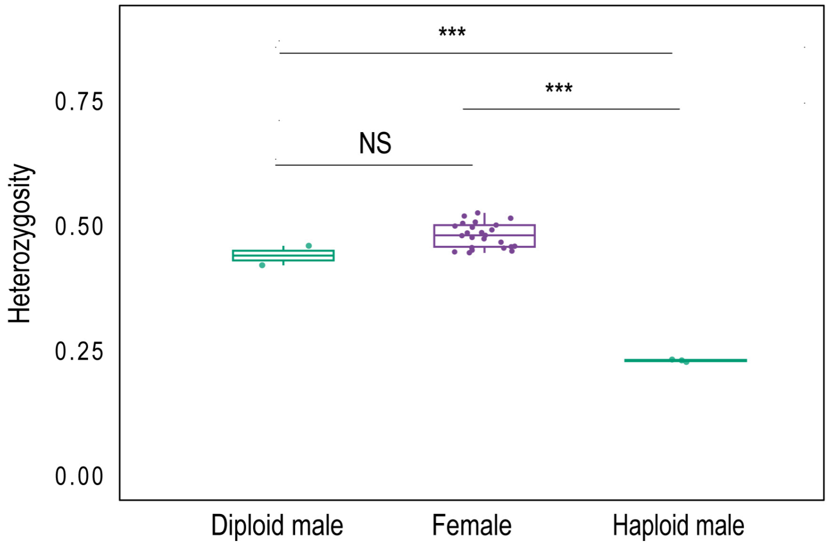

Figure S7. Genome-wide heterozygosity distinguishes ploidy in *Vespa mandarinia* individuals collected in the USA.

Each dot represents the genome-wide average heterozygosity of a single individual based on whole-genome sequencing. The dataset includes 25 females, 2 diploid males (SRR27733288 and SRR27733289), and three haploid males (SRR27733290, SRR27733372, SRR27733373). Male ploidy was inferred based on heterozygosity levels (Kruskal-Wallis test followed by pairwise Wilcoxon test with Bonferroni–Holm correction, ***=$P < 0.001$, NS = $P > 0.05$). Data reanalysed from (11); BioProject PRJNA1069611.

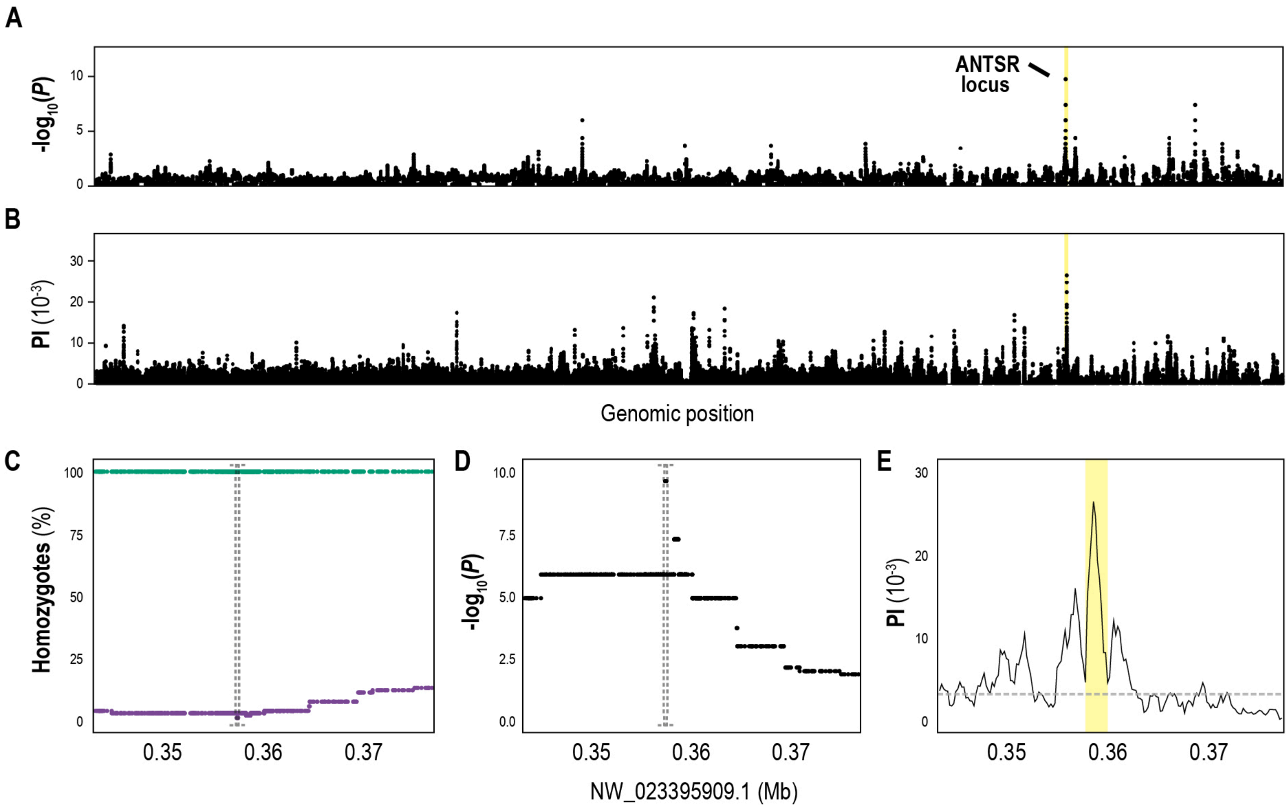

Figure S8. Genomic signature of the candidate ANTSR locus in *Vespa mandarinia*.

A. Comparison of SNP heterozygosity between females (N = 108) and diploid males (N = 2; Figure S7) from Taylor et al. (11); BioProject PRJNA1069611. Negative log10-transformed P-values from two-proportion Z-tests are plotted along genomic coordinates. **B.** Nucleotide diversity (PI) in 1-kb windows across the *V. mandarinia* genome based on 108 field-collected females (11). **C.** Homozygosity levels in females and diploid males (same samples as A), shown as the percentage of individuals with a run of homozygosity at each SNP position. In the multi-allelic sex determining region, 107 out of 108 females were heterozygous, whereas both diploid males were homozygous (dashed box; NW_023395909.1: 358,107–358,221). **D.** Close-up of panel B. **E.** Close-up of panel A. The highly polymorphic region of the ANTSR locus, defined as the region where all windows rank within the top 1% most diverse in the genome (grey dashed line), is highlighted in yellow (NW_023395909.1: 358,001–361,201).

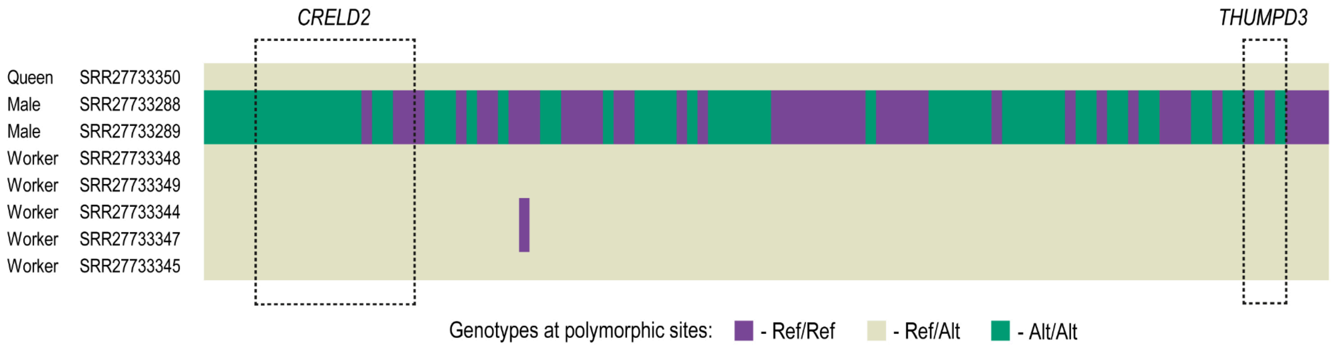

Figure S9. Genotype patterns at the *CRELD2–THUMPD3* region in the *Vespa mandarinia* family US_3.

Comparison of SNP genotypes for eight individuals from a single *V. mandarinia* family in the USA (US_3), spanning the *CRELD2–THUMPD3* region (NW_023395909.1: 355,991–381,931). Each row represents an individual, and each column corresponds to a SNP position; genotypes are color-coded by homozygosity or heterozygosity for the reference and alternative alleles. Gene boundaries for *CRELD2* and *THUMPD3* are indicated. The dataset includes one queen, five worker daughters, and two diploid male offspring (SRR27733288 and SRR27733289; see also Figure S7). The observed genotype pattern suggests that the queen mated with a male sharing one of its two haplotypes at this region, producing homozygous diploid males and heterozygous female workers. Fisher's Exact Test, $P = 0.037$. Data reanalysed from Taylor et al. (11) (BioProject PRJNA1069611).

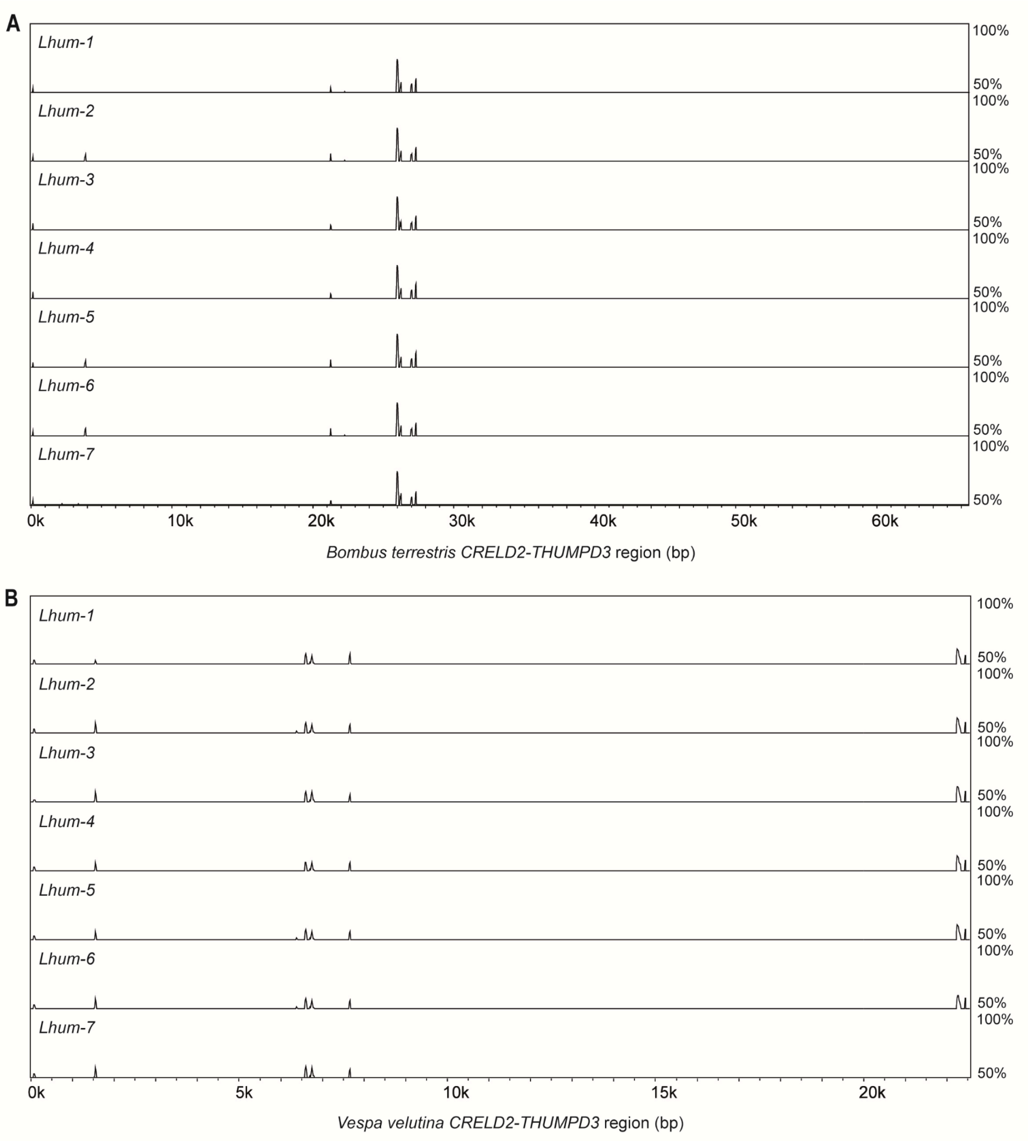

Figure S10. Sequence similarity between the seven haplotypes from the sex-determining polymorphic region of the ANTSR locus in *Linepithema humile (Lhum-1* to *Lhum-7*) and the assemblies of *Bombus terrestris* and *Vespa velutina nigrithorax.*

A. Comparison with the haploid RefSeq-annotated genome assembly of *B. terrestris* (iyBomTerr1.2) spanning the *CRELD2–THUMPD3* region. **B.** Comparison with the haploid RefSeq-annotated genome assembly of *V. velutina nigrithorax* (iVesVel2.1) covering the same region.

The *L. humile* haplotypes were assembled *de novo* from male short-read data (12) and aligned to the reference genomes using LAGAN (13) within the mVISTA suite (https://genome.lbl.gov/vista/). Conservation was computed in 100-bp windows, with the x-axis limited to 50–100% sequence identity. All regions fall below the default 70% conservation threshold, except for a short segment conserved across all haplotypes in both ants and bees. No *L. humile* haplotype shows higher similarity to either *B. terrestris* or *V. velutina nigrithorax*, suggesting the absence of trans-species polymorphism (2).

Tables

Table S1. Positional information for the *CRELD2–THUMPD3* genomic region across Hymenoptera

Coordinates indicate the genomic positions of *CRELD2*, the candidate sex-determining polymorphic region of the ANTSR locus, the putative orthologs of lncRNA *ANTSR,* and *THUMPD3* orthologs in each reference genome. The position of the candidate sex-determining polymorphic region was defined based on the three most heterozygous 5 kb windows (calculated with 1 kb steps) within the heterozygosity peak observed in females (Figure 1 and S1). For *B. terrestris*, *V. mandarinia*, and *V. velutina nigrithorax*, PI analyses refined the region to genomic intervals in which all windows rank among the top 1% most diverse in the genome; we also report these values. In *L. humile*, the region had previously been delineated by Pan et al. (12), and we also report this value. The precise position of lncRNA *ANTSR* is ambiguous in nearly all species, except for *Linepithema humile*, due to technical limitations: *ANTSR* is primarily expressed in early embryos and lacks a poly(A) tail, which prevents its detection by standard mRNA sequencing protocols (12). When at least five spliced reads with consensus splice sites were detected in the NCBI genome browser RNA-seq track, but no gene annotation was available, the coordinates of the corresponding intronic region are reported. See also Table footnotes below.

**Species Classification (Genome accession)**	**Chromosome**	***CRELD2***	**candidate sex-determining region**	**(putative) lncRNA *ANTSR***	***THUMPD3***
Apis cerana<br>Anthophila (Aculeata)<br>(GCF_029169275.1)<br>***csd* gene**	NC_083853.1	10335831-<br>10337726	N.A.	10327889-<br>10332351<br><br>10325516-<br>10327248	10302372-<br>10304896
Apis mellifera<br>Anthophila (Aculeata)<br>(GCF_003254395.2)<br>***csd* gene**	NC_037639.1	10413001-<br>10414723	N.A.	10408977-<br>10409725	10380850-<br>10383258
Bombus pascuorum<br>Anthophila (Aculeata)<br>(GCF_905332965.1)<br>***ANTSR*^{CAN}**	NC_083493.1	9935256-<br>9937139	9938001-<br>9945001	22 reads:<br>9987953-<br>9989593	10005120-<br>10007144
Bombus terrestris<br>Anthophila (Aculeata)<br>(GCF_910591885.1)<br>***ANTSR*^{VAL}**	NC_063270.1	16281875-<br>16283701	16274001-<br>16281000<br>(16278001-<br>16282201,<br>based on PI)	16256403-<br>16258912<br><br>16225130-<br>16227281	16217168-<br>16219102
Megachile willughbiella<br>Anthophila (Aculeata)<br>(GCA_945859595.2)<br>***ANTSR*^{CAN}, BLAST**	OX243803.1	7972575-<br>7973771	7968001-<br>7975001	N.A.	7922980-<br>7923608
Osmia bicornis<br>Anthophila (Aculeata)<br>(GCF_907164935.1)	NC_060216.1	14408694-<br>14410328	N.A.	14404739-<br>14405609	14380003-<br>14381978

Table S1 (continued).

**Species**<br>**Classification**<br>**(Genome accession)**	**Chromosome**	***CRELD2***	**candidate sex-determining region**	**(putative) lncRNA *ANTSR***	***THUMPD3***
Colletes gigas<br>Colletidae (Aculeata)<br>(GCF_013123115.1)<br>***ANTSR*CAN**	NW_025106639.1	3085318-3087859	3087001-3094000	N.A.	3126411-3128395
Hylaeus volcanicus<br>Colletidae (Aculeata)<br>(GCF_026283585.1)	NC_071978.1	22941173-22943861	N.A.	36 reads: 22921372-22926179	22889507-22893523
Nomia melanderi<br>Halictidae (Aculeata)<br>(GCF_003710045.2)<br>***ANTSR*CAN**	NW_022339984.1	969870-972118	958001-965000	N.A.	913894-916762
Cerceris rybyensis<br>Crabronidae (Aculeata)<br>(GCA_910591515.1)<br>***ANTSR*CAN, BLAST**	OU342792.1	23071787-23072584	23063001-23070000	N.A.	22997967-22998433
Cardiocondyla obscurior<br>Formicidae (Aculeata)<br>(GCF_019399895.1)<br>**Non-CSD, BLAST**	NC_091878.1 (*CRELD2*)<br>NC_091879.1 (*THUMPD3*)	4779605-4783177	N.A.	N.A.	1979983-1981896
Linepithema humile<br>Formicidae (Aculeata)<br>(GCF_040581485.1)<br>***ANTSR*VAL**	NC_090131.1	26518974-26520848	26527001-26534000 (26529007-26534051 in Pan et al.)	26527990-26541681	26570317-26572617
Monomorium pharaonis<br>Formicidae (Aculeata)<br>(GCF_013373865.1)<br>**Non-CSD**	NC_050476.1	7641843-7643671	N.A.	7665663-7694845	7702554-7704803
Ooceraea biroi<br>Formicidae (Aculeata)<br>(GCF_003672135.1)<br>***ANTSR*VAL**	NC_039509.1	1727256-1729679	1717001-1724000	1687395-1693282	1675932-1678296
Solenopsis invicta<br>Formicidae (Aculeata)<br>(GCF_016802725.1)<br>***ANTSR*CAN**	NC_052666.1	32521097-32524775	32526001-32533000	32563531-32565667	32576650-32579036
Vollenhovia emeryi<br>Formicidae (Aculeata)<br>(GCF_000949405.1)<br>***ANTSR*CAN**	NW_011967112.1	131243-133928	N.A.	N.A.	59541-62114

Table S1 (continued).

**Species**<br>**Classification**<br>**(Genome accession)**	**Chromosome**	***CRELD2***	**candidate sex-determining region**	**(putative) lncRNA *ANTSR***	***THUMPD3***
Cryptocheilus praepositus<br>Pompilidae (Aculeata)<br>(GCA_033815515.1)<br>***ANTSR*^CAN^, BLAST**	JAWWQZ010000035.1	2467568-2468667	2450001-2457000	N.A.	2416717-2418030
Priocnemis perturbator<br>Pompilidae (Aculeata)<br>(GCA_963942575.1)<br>**BLAST**	OZ012645.1	13959727-13960841	N.A.	N.A.	14005490-14006682
Tiphia femorata<br>Pompilidae (Aculeata)<br>(GCA_944319695.1)<br>***ANTSR*^CAN^, BLAST**	OX090903.1	13626524-13627626	13621001-13628000	N.A.	13577640-13578367
Polistes fuscatus<br>Vespidae (Aculeata)<br>(GCF_010416935.1)<br>***ANTSR*^CAN^**	NW_025113140.1	5602382-5603923	5606001-5613000	N.A.	5628915-5631077
Vespa crabro<br>Vespidae (Aculeata)<br>(GCF_910589235.1)<br>***ANTSR*^CAN^**	NC_060977.1	971852-973699	972001-979000	977299-978531<br><br>984882-985309	991126-993048
Vespa mandarinia<br>Vespidae (Aculeata)<br>(GCF_014083535.1)<br>***ANTSR*^VAL^**	NW_023395909.1	356133-357991	356001-363000 (358001–361201 based on PI)	11 reads: 364323-369360	375720-377901
Vespa velutina nigrithorax<br>Vespidae (Aculeata)<br>(GCF_912470025.1)<br>***ANTSR*^VAL^**	NC_062210.1	3365834-3369044	3361001-3367000 (3362601-3366601 based on PI)	6 reads: 3361538-3361683	3346487-3348473
Vespula vulgaris<br>Vespidae (Aculeata)<br>(GCF_905475345.1)<br>***ANTSR*^CAN^**	NC_066608.1	347119-348850	348001-355000	352944-364185	366600-368830
Hedychridium roseum<br>Chrysidoidea (Aculeata)<br>(GCA_963989215.1)<br>**BLAST**	OZ022304.1	11209614-11210448	N.A.	N.A.	11232292-11233686
Sclerodermus 'alternatusi'<br>Chrysidoidea (Aculeata)<br>(GCA_037305995.1)<br>**BLAST**	JBBEEM010000025.1	164928-165758	N.A.	N.A.	139605-140935

Table S1 (continued).

Species<br>Classification<br>(Genome accession)	Chromosome	*CRELD2*	*THUMPD3*
Aphidius gifuensis<br>Braconidae (Apocrita)<br>(GCF_014905175.1)	NC_057790.1<br>(*CRELD2*)<br>NC_057792.1<br>(*THUMPD3*)	22557401-<br>22559326	17537566-<br>17539419
Cotesia glomerata<br>Braconidae (Apocrita)<br>(GCF_020080835.1)	NC_058164.1	422626-<br>425237	6460686-<br>6463604
Fopius arisanus<br>Braconidae (Apocrita)<br>(GCF_000806365.1)	NW_011887755.1	844761-<br>846541	986207-<br>988057
Lysiphlebus fabarum<br>Braconidae (Apocrita)<br>(GCA_011426435.1)<br>**BLAST**	WSYQ01001071.1<br>(*CRELD2*)<br>WSYQ01001077.1<br>(*THUMPD3*)	241623-<br>243025	204304-<br>205623
Microplitis demolitor<br>Braconidae (Apocrita)<br>(GCF_026212275.2)	NC_068551.1	22220823-<br>22222722	17810678-<br>17813330
Belonocnema kinseyi<br>Cynipidae (Apocrita)<br>(GCF_010883055.1)<br>**BLAST**	NC_046663.1<br>(*CRELD2*)	113529810-<br>113548991	N.A
Leptopilina boulardi<br>Figitidae (Apocrita)<br>(GCF_019393585.1)	NW_02613796.11<br>(*CRELD2*)<br>NW_026137956.1<br>(*THUMPD3*)	4238808-<br>4244801	428600-<br>432569
Leptopilina heterotoma<br>Figitidae (Apocrita)<br>(GCF_015476425.1)	NW_025110882.1<br>(*CRELD2*)<br>NW_025111040.1<br>(*THUMPD3*)	432154-<br>435505	1422846-<br>1424968
Venturia canescens<br>Ichneumonidae (Apocrita)<br>(GCF_019457755.1)	NC_057428.1	18958311-<br>18961205	22321472-<br>22323693

Table S1 (continued).

**Species**<br>**Classification**<br>**(Genome accession)**	**Chromosome**	***CRELD2***	***THUMPD3***
Nasonia vitripennis<br>Pteromalidae (Apocrita)<br>(GCF_000002325.3)<br>**Non-CSD, BLAST**	NC_045759.1<br>(*CRELD2*)	4795003-<br>4797139	N.A
Diprion similis<br>Diprionidae (Symphyta)<br>(GCF_021155765.1)	NC_060114.1	21933320-<br>21935918	20526566-<br>20528595
Neodiprion lecontei<br>Diprionidae (Symphyta)<br>(GCF_021901455.1)	NC_060264.1	35986250-<br>35989221	34541825-<br>34543768
Neodiprion pinetum<br>Diprionidae (Symphyta)<br>(GCF_021155775.2)<br>**BLAST**	NC_060236.1<br>(*CRELD2*)<br>NC_060232.1<br>(*THUMPD3*)	35348686-<br>35351656	5145701-<br>5149948
Athalia rosae<br>Tenthredinidae (Symphyta)<br>(GCF_917208135.1)	NC_064030.1	14847629-<br>14850303	16980763-<br>16982737
Euura cinereae<br>Tenthredinidae (Symphyta)<br>(GCA_018420115.1)<br>**BLAST**	JAFFZH010013383.1<br>(*CRELD2*)<br>JAFFZH010010118.1<br>(*THUMPD3*)	5718-<br>7341	3705-<br>5529
Euura lappo<br>Tenthredinidae (Symphyta)<br>(GCA_018257835.1)<br>**BLAST**	JAEUYN010002191.1<br>(*CRELD2*)<br>JAEUYN010002145.1<br>(*THUMPD3*)	6893-<br>7996	159181-<br>161272

Table footnotes: ANTSR^{VAL}: Role of the ANTSR locus in sex determination validated through genetic mapping from (12, 14) and this study. ***ANTSR^{CAN}***: Candidate role of the ANTSR locus in sex determination inferred from patterns of female-specific heterozygosity (this study). We also include in this list the ant *Vollenhovia emeryi*, where the ANTSR locus is part of a large QTL associated with sex differences (15). ***csd* gene**: Honeybee species that rely on complementary sex determination (CSD) and use the recently evolved, lineage-specific protein-coding gene *csd* as the primary sex-determining locus. **Non-CSD**: Species that do not rely on complementary sex determination (Text S1). **BLAST**: Gene positions for *CRELD2* and *THUMPD3* were determined via BLASTn using annotated transcripts from *L. humile* or closely related species.

Table S2. NCBI accessions of female samples from Aculeata used in heterozygosity analyses.

Heterozygosity in the candidate location of the polymorphic sex-determining region of the ANTSR locus was examined in 17 species beyond *Linepithema humile*. Four negative control species, known not to rely on heterozygosity at the ANTSR locus for sex determination (see Text S1), are highlighted in grey. Accessions correspond to the female individuals used for whole-genome sequencing analyses.

Species	(Super)family	Accession
Apis cerana	Anthophila	SRR27281405
Apis mellifera	Anthophila	SRR28865845
Bombus pascuorum	Anthophila	ERR6054546
Bombus terrestris	Anthophila	ERR7800057
Cardiocondyla obscurior	Formicidae	SRR13108031
Cerceris rybyensis	Crabronidae	ERR6436387
Colletes gigas	Colletidae	SRR16214293
Cryptocheilus praepositus	Pompiloidea	SRR22857819
Hedychridium roseum	Chrysidoidea	ERR12085104
Linepithema humile	Formicidae	SRR29344957
Megachile willughbiella	Anthophila	ERR9854837
Monomorium pharaonis	Formicidae	SRR16493416
Nomia melanderi	Halictidae	SRR7977585
Ooceraea biroi	Formicidae	SRR23947734
Polistes fuscatus	Vespidae	SRR15915334
Sclerodermus alternatusi	Chrysidoidea	SRR28335762
Solenopsis invicta	Formicidae	SRR9127698
Tiphia femorata	Tiphioidea	ERR9467445
Vespa crabro	Vespidae	ERR6054856
Vespa mandarinia	Vespidae	SRR27733390
Vespa velutina nigrithorax	Vespidae	Sample ED07 (this study)
Vespula vulgaris	Vespidae	ERR6054670

Table S3. Monitoring of offspring production in *Bombus terrestris* colonies headed by sib-mated queens.

Offspring were monitored for three months after queens resumed egg laying. Queens were classified as early-male-producing if they produced >1 male within this period, or worker-producing if they produced no males (or only a single male). Nine early-male-producing and one worker-producing colonies were selected for sequencing. The total number of worker females and males sequenced per colony is indicated. Three haploid males were sequenced as controls (*).

			Total number of offspring after 3 months		Genome sequenced	
**Grandmother**	**Mother**	**Phenotype**	**Workers**	**Males**	**Males**	**Workers**
C	C71	Early-male-producing	27	24	4	4
C	C72	Worker-producing	35	1		
C	C73	Worker-producing	52	0		
C	C74	Worker-producing	51	0		
C	C75	Early-male-producing	16	19	4	4
C	C76	Early-male-producing	11	8		
C	C77	Early-male-producing	13	11	4	4
C	C78	Worker-producing	10	0		
C	C80	Worker-producing	22	1	1*	
C	C81	Early-male-producing	2	4		
C	C82	Early-male-producing	21	13	4	4
C	C83	Early-male-producing	7	20		
C	C84	Early-male-producing	22	16	3	4
C	C85	Early-male-producing	24	27	3	4
C	C86	Worker-producing	44	1		
C	C87	Early-male-producing	5	7		
C	C90	Worker-producing	25	0		
C	C91	Worker-producing	25	0		
C	C92	Worker-producing	14	0		
C	C93	Early-male-producing	8	12		
C	C94	Worker-producing	31	0		
U	U61	Early-male-producing	7	7		
U	U62	Early-male-producing	5	7		
U	U65	Worker-producing	2	0		
U	U66	Early-male-producing	4	6		
U	U67	Early-male-producing	27	30	1	2
U	U68	Early-male-producing	13	15		
U	U70	Early-male-producing	8	10	4	4
U	U73	Worker-producing	47	0		
U	U74	Early-male-producing	11	19	2 + 2*	4

Table S4. Samples from inbred *Bombus terrestris* laboratory crosses used for whole-genome sequencing.

Samples were obtained from sib-mated crosses and include worker females, diploid males, and haploid males used as controls (*). Sequencing coverage (Gb) and effective read depth (average number of reads per position) after PCR duplicate removal are indicated for each individual.

Sample	Mother	Caste/Sex	Coverage	Effective read depth
C71M1	C71	Male	7.68	13.25
C71M2	C71	Male	4.27	6.92
C71M3	C71	Male	10.03	10.36
C71M4	C71	Male	8.16	11.77
C71W1	C71	Worker	13.30	19.43
C71W2	C71	Worker	6.06	8.89
C71W3	C71	Worker	9.05	12.58
C71W4	C71	Worker	8.55	12.49
C75M1	C75	Male	4.79	6.01
C75M2	C75	Male	5.51	10.81
C75M3	C75	Male	6.78	10.83
C75M4	C75	Male	4.87	8.30
C75W1	C75	Worker	12.73	17.17
C75W2	C75	Worker	6.81	9.87
C75W3	C75	Worker	6.87	10.63
C75W4	C75	Worker	7.27	10.43
C77M1	C77	Male	4.57	7.45
C77M2	C77	Male	7.17	11.16
C77M3	C77	Male	6.39	9.14
C77M4	C77	Male	8.12	10.40
C77W1	C77	Worker	5.22	8.27
C77W2	C77	Worker	4.57	6.32
C77W3	C77	Worker	3.56	5.78
C77W4	C77	Worker	6.10	8.85
C80M1	C80	Male *	6.89	10.08
C80W1	C80	Worker	6.24	7.85
C80W2	C80	Worker	4.64	6.75
C80W3	C80	Worker	5.97	8.86
C80W4	C80	Worker	6.08	9.41
C82M1	C82	Male	9.69	15.85
C82M2	C82	Male	8.73	10.08
C82M3	C82	Male	4.27	5.56
C82M4	C82	Male	3.52	4.64
C82W1	C82	Worker	15.40	17.34
C82W2	C82	Worker	5.28	6.28
C82W3	C82	Worker	4.49	6.86
C82W4	C82	Worker	8.82	12.43
C84M2	C84	Male	10.64	12.90

Table S4 (continued).

Sample	Mother	Caste/Sex	Coverage	Effective read depth
C84M3	C84	Male	3.61	4.50
C84M4	C84	Male	10.52	13.03
C84W1	C84	Worker	7.56	13.02
C84W2	C84	Worker	6.38	7.62
C84W3	C84	Worker	5.26	8.27
C84W4	C84	Worker	11.18	14.41
C85M2	C85	Male	5.23	7.25
C85M3	C85	Male	5.34	8.46
C85M4	C85	Male	7.10	11.19
C85W1	C85	Worker	4.11	4.57
C85W2	C85	Worker	3.70	4.55
C85W3	C85	Worker	10.10	15.03
C85W4	C85	Worker	5.89	10.04
U67M4	U67	Male	17.84	18.42
U67W3	U67	Worker	7.38	13.17
U67W4	U67	Worker	5.67	10.20
U70M1	U70	Male	3.79	7.22
U70M2	U70	Male	10.71	12.56
U70M3	U70	Male	8.24	13.52
U70M4	U70	Male	7.76	11.71
U70W1	U70	Worker	11.61	13.96
U70W2	U70	Worker	8.26	11.23
U70W3	U70	Worker	3.23	6.01
U70W4	U70	Worker	8.44	13.62
U74M1	U74	Male	2.89	5.45
U74M2	U74	Male	7.37	13.66
U74M3	U74	Male *	7.25	11.79
U74M4	U74	Male *	6.92	11.61
U74W1	U74	Worker	10.83	14.27
U74W2	U74	Worker	5.86	9.01
U74W3	U74	Worker	4.39	7.96
U74W4	U74	Worker	6.36	10.66

Table S5. Inference of sex-locus haplotypes in field-collected *Bombus terrestris* samples.

Haplotypes were inferred from whole-genome data reanalysed from (9, 10). For each sample, the geographic origin and assigned haplotype (corresponding to the clades shown in Figure 2E) are indicated.

Accession	Individual	Island population	Haplotype
SRR11647856	2014_Bter_A_Male_8_141_L003L008	Great Britain	A
SRR11862157	2014_Bter_P_Male_11_204_L008L006	Great Britain	A
SRR11745184	2014_Bter_A_Male_29_649_L003L008	Great Britain	B
SRR11780512	2014_Bter_M_Male_5_84_L008L006	Great Britain	B
SRR11861506	2014_Bter_P_Male_11_201_L003L008	Great Britain	B
SRR11879878	2014_Bter_P_Male_13_227_L003L008	Great Britain	B
SRR12161711	2014_Bter_P_Male_15_286_L008L006	Great Britain	B
SRR12168508	2014_Bter_P_Male_22_447_L003L008	Great Britain	B
SRR12169215	2014_Bter_P_Male_24_477_L003L008	Great Britain	B
SRR11658994	2014_Bter_A_Male_16_309_L008L006	Great Britain	C
SRR12180931	2014_Bter_A_Male_18_359_L008L006	Great Britain	C
SRR11640395	2014_Bter_A_Male_7_119_L003L008	Great Britain	C
SRR11777740	2014_Bter_M_Male_4_62_L003L008	Great Britain	C
SRR11802589	2014_Bter_P_Male_1_11_L003L008	Great Britain	C
SRR11885031	2014_Bter_P_Male_15_278_L003L008	Great Britain	C
SRR12168699	2014_Bter_P_Male_23_470_L003L008	Great Britain	C
SRR12171173	2014_Bter_P_Male_28_633_L003L008	Great Britain	C
SRR11773638	2014_Bter_M_Male_2_25_L003L008	Great Britain	D
SRR18329090	2014_Bter_M_Male_6_91_L008L006	Great Britain	D
SRR12165042	2014_Bter_P_Male_22_446_L008L006	Great Britain	D
SRR11659726	2014_Bter_A_Male_17_325_L003L008	Great Britain	E
SRR12179650	2014_Bter_A_Male_19_499_L008L006	Great Britain	E
SRR12174541	2014_Bter_A_Male_21_408_L008L006	Great Britain	E
SRR11745230	2014_Bter_A_Male_29_650_L008L006	Great Britain	E
SRR12173003	2014_Bter_P_Male_1_13_L008L006	Great Britain	E
SRR11654235	2014_Bter_P_Male_16_311_L003L008	Great Britain	E
SRR11803984	2014_Bter_P_Male_9_158_L003L008	Great Britain	E
SRR12171313	2014_Bter_P_Male_9_164_L008L006	Great Britain	E
SRR11665914	2014_Bter_A_Male_17_339_L008L006	Great Britain	F
SRR11742810	2014_Bter_A_Male_21_400_L003L008	Great Britain	F
SRR11637993	2014_Bter_A_Male_3_40_L008L006	Great Britain	F
SRR11780128	2014_Bter_M_Male_4_63_L008L006	Great Britain	F
SRR11881496	2014_Bter_P_Male_14_260_L003L008	Great Britain	F
SRR12170585	2014_Bter_P_Male_25_519_L003L008	Great Britain	F
SRR11668399	2014_Bter_A_Male_18_353_L003L008	Great Britain	G
SRR11722087	2014_Bter_A_Male_19_503_L008L006	Great Britain	G
SRR11729941	2014_Bter_A_Male_19_509_L008L006	Great Britain	G
SRR11744704	2014_Bter_A_Male_28_632_L008L006	Great Britain	G
SRR11746805	2014_Bter_A_Male_40_705_L008L006	Great Britain	G
SRR11871334	2014_Bter_P_Male_12_638_L003L008	Great Britain	G

Table S5 (continued).

Accession	Individual	Island population	Haplotype
SRR11880654	2014_Bter_P_Male_13_237_L008L006	Great Britain	G
SRR11883556	2014_Bter_P_Male_14_263_L008L006	Great Britain	G
SRR12170707	2014_Bter_P_Male_26_572_L003L008	Great Britain	G
SRR11711132	2014_Bter_A_Male_19_495_L003L008	Great Britain	H
SRR11744605	2014_Bter_A_Male_27_600_L003L008	Great Britain	H
SRR11625411	2014_Bter_A_Male_3_39_L003L008	Great Britain	H
SRR11747770	2014_Bter_A_Male_40_719_L003L008	Great Britain	H
SRR11649676	2014_Bter_A_Male_8_149_L008L006	Great Britain	H
SRR11775825	2014_Bter_M_Male_2_34_L008L006	Great Britain	H
SRR11780662	2014_Bter_M_Male_6_92_L008L006	Great Britain	H
SRR12170781	2014_Bter_P_Male_26_588_L008L006	Great Britain	H
SRR23681995	2018_Bter_CV_Male_MU_17_L1	Ireland	A
SRR23649187	2018_Bter_DB_Male_MU_44_L1	Ireland	A
SRR23675069	2018_Bter_KY_Male_MU_26_L1	Ireland	A
SRR23651148	2018_Bter_LM_Male_MU_32_L1	Ireland	A
SRR23684823	2018_Bter_OF_Male_MU_06_L1	Ireland	A
SRR23684822	2018_Bter_WK_Male_MU_03_L1	Ireland	A
SRR23675071	2018_Bter_WX_Male_MU_28_L2	Ireland	A
SRR23651150	2018_Bter_FM_Male_MU_35_L1	Ireland	B
SRR23675066	2018_Bter_WM_Male_MU_22_L1	Ireland	B
SRR23684824	2018_Bter_WX_Male_MU_07_L2L4	Ireland	B
SRR23675072	2018_Bter_WK_Male_MU_29_L2	Ireland	C
SRR23684825	2018_Bter_GW_Male_MU_08_L1	Ireland	D
SRR23684826	2018_Bter_GW_Male_MU_09_L1	Ireland	D
SRR23681993	2018_Bter_KD_Male_MU_10_L1	Ireland	D
SRR23675064	2018_Bter_KD_Male_MU_20_L1	Ireland	D
SRR23675065	2018_Bter_KD_Male_MU_21_L1	Ireland	D
SRR23675067	2018_Bter_MT_Male_MU_23_L1	Ireland	D
SRR23649183	2018_Bter_WK_Male_MU_40_L1	Ireland	D
SRR23649188	2018_Bter_WX_Male_MU_45_L1	Ireland	D
SRR23681996	2018_Bter_AT_Male_MU_18_L1	Ireland	E
SRR23681994	2018_Bter_CK_Male_MU_12_L1	Ireland	E
SRR23675070	2018_Bter_CK_Male_MU_27_L1	Ireland	E
SRR23649184	2018_Bter_DB_Male_MU_41_L1	Ireland	E
SRR23649185	2018_Bter_KY_Male_MU_42_L2	Ireland	E
SRR23649186	2018_Bter_KY_Male_MU_43_L1	Ireland	E
SRR23647780	2018_Bter_CL_Male_MU_46_L1	Ireland	G
SRR23651152	2018_Bter_FM_Male_MU_38_L2	Ireland	G
SRR23651151	2018_Bter_KD_Male_MU_36_L2	Ireland	G
SRR23651149	2018_Bter_LH_Male_MU_33_L1	Ireland	G
SRR23681997	2018_Bter_AT_Male_MU_19_L1	Ireland	H
SRR23684821	2018_Bter_CW_Male_MU_01_L1	Ireland	H
SRR23675068	2018_Bter_KY_Male_MU_25_L1	Ireland	H
SRR23651153	2018_Bter_WM_Male_MU_39_L2	Ireland	H

Table S6. ***Vespa velutina nigrithorax*** **samples collected in France and used for whole-genome sequencing.**

Samples were collected by E. Darrouzet and colleagues and used for whole-genome sequencing analyses. Sample identifiers, collection localities, sequencing details, and inferred genotypes at the candidate polymorphic sex-determining region of the ANTSR locus are provided. Diploid male status was confirmed by microsatellite genotyping [32].

Sample	Nest	Caste	Coverage	Effective read depth	*ANTSR locus*
A01	A	Diploid male	3.94	2.38	D/D
B02	B	Diploid male	2.06	2.31	A/A
C1Y1	C01	Diploid male	3.24	4.95	A/A
C2Y2	C02	Diploid male	5.22	6.34	C/C
C3Y1	C03	Diploid male	13.46	23.48	A/A
C4Y1	C04	Diploid male	10.08	18.01	B/B
C6Y1	C06	Diploid male	11.28	21.15	A/A
AW21	A	Worker	5.22	5.77	A/D
BW19	B	Worker	2.43	5.20	A/C
CW05	C	Worker	5.15	10.68	A/D
ED01	ED01	Worker	5.10	9.11	A/D
ED02	ED02	Worker	4.00	6.99	A/C
ED03	ED03	Worker	4.29	8.03	B/C
ED04	ED04	Worker	4.09	7.19	A/B
ED05	ED05	Worker	3.73	6.03	C/D
ED06	ED06	Worker	3.92	5.89	C/D
ED07	ED07	Worker	10.68	12.92	C/D
ED08	ED08	Worker	3.51	5.30	A/C
ED09	ED09	Worker	5.15	8.39	B/D
ED10	ED10	Worker	4.91	9.25	B/C
ED11	ED11	Worker	4.31	6.31	B/C
ED12	ED12	Worker	5.30	8.25	B/D
ED13	ED13	Worker	11.82	11.68	A/D
ED14	ED14	Worker	2.55	3.76	A/D
ED15	ED15	Worker	3.35	5.32	A/C
ED16	ED16	Worker	2.85	4.71	B/C
ED17	ED17	Worker	2.90	4.04	A/C
ED18	ED18	Worker	4.46	6.83	A/B
ED19	ED19	Worker	3.25	4.96	A/D
ED20	ED20	Worker	4.45	4.73	B/C
ED21	ED21	Worker	8.36	12.36	B/D
ED22	ED22	Worker	3.57	5.56	A/D

Table S7. Variant calling, filtering, and PLINK parameters used for population genomic analyses

Parameter adjustments were implemented in each species to account for differences in genome-wide diversity, repeat content, and sampling strategy, thereby maximizing the signal-to-noise ratio to a comparable level among species. Biallelic SNPs with sequencing depth between 0.5× and 2× the genome-wide mean were retained. To reduce alignment artefacts, only variants with a mean mapping quality ≥60 for both reference and alternate alleles were retained in *Vespa velutina nigrithorax* and *Bombus terrestris*. Sites heterozygous in haploid *B. terrestris* males were excluded from the Pi analyses..

**Species**	**Comparison of males and females**	**Genetic diversity (Pi)**
Vespa velutina nigrithorax<br>GCF_912470025.1	7 diploid males and 25 worker females (Table S6)<br><br>*Freebayes: --haplotype-length 0*<br><br>*Hard-masking: Yes*<br><br>*VCFtools: --remove-indels --minQ 30 --maf 0.1 --mac 3 --min-alleles 2 --max-alleles 2 --min-meanDP 2 --max-meanDP 20*<br><br>*BCFtools: view -i 'INFO/MQM>=60 && INFO/MQMR>=60'*<br><br>*Plink: --homozyg-window-snp 50 --homozyg-snp 50 --homozyg-gap 5000 --homozyg-window-missing 35 --homozyg-kb 20 --homozyg-density 1000 --homozyg-window-het 1*	25 unrelated female workers (Table S6)<br><br>*Freebayes: --haplotype-length 0*<br><br>*Hard-masking: Yes*<br><br>*VCFtools: --remove-indels --minQ 30 --maf 0.1 --mac 3 --min-alleles 2 --max-alleles 2 --min-meanDP 2 --max-meanDP 20*<br><br>*BCFtools: view -i 'INFO/MQM>=60 && INFO/MQMR>=60'*
Bombus terrestris<br>GCF_910591885.1	29 diploid males and 34 worker females (Table S4)<br><br>*Freebayes: --haplotype-length 0*<br><br>*Hard-masking: Yes*<br><br>*VCFtools: --remove-indels --minQ 30 --maf 0.1 --mac 3 --min-alleles 2 --max-alleles 2 --min-meanDP 2 --max-meanDP 12*<br><br>*BCFtools: view -i 'INFO/MQM>=60 && INFO/MQMR>=60'*<br><br>*Plink: --homozyg-window-snp 10 --homozyg-snp 20 --homozyg-gap 200 --homozyg-window-missing 8 --homozyg-kb 50 --homozyg-density 10 --homozyg-window-het 2*	84 unrelated haploid males (Table S5)<br><br>*Freebayes: -use-best-n-alleles 4 --haplotype-length 0 --min-base-quality 3 --min-mapping-quality 1 --min-alternate-frac 0.25 --min-coverage 1*<br><br>*Hard-masking: Yes*<br><br>*VCFtools: --remove-indels --minQ20 --maf 0.01 --mac 5 --min-meanDP 2 --max-meanDP 15 --min-alleles 2 --max-alleles 2*<br><br>*BCFtools: view -e 'GT="0/1" | GT="1/0"'*

Table S7 (continued).

**Species**	**Comparison of males and females**	**Genetic diversity (Pi)**
Vespa mandarinia<br>GCF_014083535.2	2 diploid males (Figure S7)<br>108 females (11)<br><br>*Freebayes: --skip-coverage 4000 --use-best-n-alleles 2 --haplotype-length 0*<br><br>*Hard-masking: No*<br><br>*VCFtools: --remove-indels --minQ 20 --maf 0.01 --mac 4 --min-alleles 2 --max-alleles 2 --min-meanDP 10 --max-meanDP 35*<br><br>*BCFtools: Not used*<br><br>*Plink: --homozyg-window-snp 10 --homozyg-snp 20 --homozyg-gap 200 --homozyg-window-missing 8 --homozyg-kb 50 --homozyg-density 10 --homozyg-window-het 2*	54 unrelated females<br>(11)<br><br>*Freebayes: --skip-coverage 4000 --use-best-n-alleles 2 --haplotype-length 0*<br><br>*Hard-masking: No*<br><br>*VCFtools: --remove-indels --minQ 20 --maf 0.01 --mac 4 --min-alleles 2 --max-alleles 2 --min-meanDP 10 --max-meanDP 35*<br><br>*BCFtools: Not used*

SI References

1. M. Beye, M. Hasselmann, M. K. Fondrk, R. E. Page, S. W. Omholt, The gene *csd* is the primary signal for sexual development in the honeybee and encodes an SR-type protein. *Cell* **114** (2003).

2. S. Cho, Z. Y. Huang, D. R. Green, D. R. Smith, J. Zhang, Evolution of the complementary sex-determination gene of honey bees: Balancing selection and trans-species polymorphisms. *Genome Research* **16**, 1366 (2006).

3. M. Hasselmann, *et al.*, Evidence for the evolutionary nascence of a novel sex determination pathway in honeybees. *Nature* **454**, 519–522 (2008).

4. M. Otte, O. Netschitailo, S. Weidtkamp-Peters, C. A. M. Seidel, M. Beye, Recognition of polymorphic Csd proteins determines sex in the honeybee. *Sci. Adv.* **9**, eadg4239 (2023).

5. A. Schrempf, S. Aron, J. Heinze, Sex determination and inbreeding depression in an ant with regular sib-mating. *Heredity (Edinb)* **97**, 75–80 (2006).

6. A. Rajakumar, *et al.*, From egg to adult: A developmental table of the ant *Monomorium pharaonis*. *J. Exp. Zool. B Mol. Dev. Evol.* **342**, 557–585 (2024).

7. M. K. Asplen, J. B. Whitfield, J. G. DE Boer, G. E. Heimpel, Ancestral state reconstruction analysis of hymenopteran sex determination mechanisms. *J. Evol. Biol.* **22**, 1762–1769 (2009).

8. Y. Zou, E. Geuverink, L. W. Beukeboom, E. C. Verhulst, L. van de Zande, A chimeric gene paternally instructs female sex determination in the haplodiploid wasp *Nasonia*. *Science* **370**, 1115–1118 (2020).

9. T. J. Colgan, *et al.*, Genomic signatures of recent adaptation in a wild bumblebee. *Mol Biol Evol* **39**, msab366 (2022).

10. S. J. Larragy, J. S. Möllmann, J. C. Stout, J. C. Carolan, T. J. Colgan, Signatures of adaptation, constraints, and potential redundancy in the canonical immune genes of a key pollinator. *Genome Biol Evol* **15**, evad039 (2023).

11. B. A. Taylor, *et al.*, Population genomics of the invasive Northern Giant Hornet *Vespa mandarinia* in North America and across its native range. *Sci. Rep.* **14**, 10803 (2024).

12. Q. Pan, H. Darras, L. Keller, LncRNA gene *ANTSR* coordinates complementary sex determination in the Argentine ant. *Sci. Adv.* **10**, eadp1532 (2024).

13. M. Brudno, *et al.*, LAGAN and Multi-LAGAN: efficient tools for large-scale multiple alignment of genomic DNA. *Genome Res.* **13**, 721–731 (2003).

14. K. D. Lacy, *et al.*, Heterozygosity at a conserved candidate sex determination locus is associated with female development in the clonal raider ant (*Ooceraea biroi*). *eLife,* 14:RP106913 (2025).

15. M. O. Miyakawa, A. S. Mikheyev, QTL mapping of sex determination loci supports an ancient pathway in ants and honey bees. *PLoS Genet.* **11**, e1005656 (2015).
